# Mining a database of single amplified genomes from Red Sea brine pool extremophiles—improving reliability of gene function prediction using a profile and pattern matching algorithm (PPMA)

**DOI:** 10.3389/fmicb.2014.00134

**Published:** 2014-04-07

**Authors:** Stefan W. Grötzinger, Intikhab Alam, Wail Ba Alawi, Vladimir B. Bajic, Ulrich Stingl, Jörg Eppinger

**Affiliations:** ^1^Division of Physical Sciences and Engineering, KAUST Catalysis Center, King Abdullah University of Science and TechnologyThuwal, Kingdom of Saudi Arabia; ^2^Division of Biological Sciences and Engineering, Computational Bioscience Research Center, King Abdullah University of Science and TechnologyThuwal, Kingdom of Saudi Arabia; ^3^Division of Biological Sciences and Engineering, Red Sea Research Center, King Abdullah University of Science and TechnologyThuwal, Kingdom of Saudi Arabia

**Keywords:** bioinformatics, single amplified genomes, halophiles, extermophile, protein sequence consensus patterns, PROSITE IDs, GO-terms, functional genomics

## Abstract

Reliable functional annotation of genomic data is the key-step in the discovery of novel enzymes. Intrinsic sequencing data quality problems of single amplified genomes (SAGs) and poor homology of novel extremophile's genomes pose significant challenges for the attribution of functions to the coding sequences identified. The anoxic deep-sea brine pools of the Red Sea are a promising source of novel enzymes with unique evolutionary adaptation. Sequencing data from Red Sea brine pool cultures and SAGs are annotated and stored in the Integrated Data Warehouse of Microbial Genomes (INDIGO) data warehouse. Low sequence homology of annotated genes (no similarity for 35% of these genes) may translate into false positives when searching for specific functions. The Profile and Pattern Matching (PPM) strategy described here was developed to eliminate false positive annotations of enzyme function before progressing to labor-intensive hyper-saline gene expression and characterization. It utilizes InterPro-derived Gene Ontology (GO)-terms (which represent enzyme function profiles) and annotated relevant PROSITE IDs (which are linked to an amino acid consensus pattern). The PPM algorithm was tested on 15 protein families, which were selected based on scientific and commercial potential. An initial list of 2577 enzyme commission (E.C.) numbers was translated into 171 GO-terms and 49 consensus patterns. A subset of INDIGO-sequences consisting of 58 SAGs from six different taxons of bacteria and archaea were selected from six different brine pool environments. Those SAGs code for 74,516 genes, which were independently scanned for the GO-terms (profile filter) and PROSITE IDs (pattern filter). Following stringent reliability filtering, the non-redundant hits (106 profile hits and 147 pattern hits) are classified as reliable, if at least two relevant descriptors (GO-terms and/or consensus patterns) are present. Scripts for annotation, as well as for the PPM algorithm, are available through the INDIGO website.

## Introduction

Discovery of extremophilic enzymes has developed into a major driver for the biotech industry. Although many industrially relevant enzymes were isolated from organisms growing at high temperature, high salt concentration, or in environments contaminated with organic solvents, significant challenges and limitations exist for bio-prospecting of extremophilic enzymes (Liszka et al., 2012). It was estimated that only as few as 0.001–0.1% of microbes in the seawater are currently cultivatable (Amann et al., 1995) and until recently the bottleneck of cultivation not only biased the view of microbial diversity but limited the appreciation of the microbial world in general (Hugenholtz and Tyson, 2008). Novel culture-independent techniques allow the identification of thousands of novel protein motifs, domains and families from different environments (Yooseph et al., 2007). Despite the vast expectations, metagenomic data have not yet lead to the expected boost of biotechnology (Chistoserdova, 2010), mostly because they suffer from short read length, a low probability to identify rare populations (below 1%) (Kunin et al., 2008), and difficulties in assembling larger contigs of genetic material for members of complex communities. Single-cell genomics (Lasken, 2007) circumvents this problem, and larger contigs from uncultured organisms can be analyzed. A major challenge in mining genomic data of uncultured organisms is a lack of homology to genes of established organisms resulting in limited reliability of gene annotation.

A promising source of novel organisms are the deep-sea anoxic brine pools in the northern part of the Red Sea, formed by tectonic shifts (Gurvich, 2006). Interstitial brine was expulsed due to tectonic movements that allowed re-dissolution of evaporitic deposits, and/or phase separation due to temperature variations (Cita, 2006; Hovland et al., 2006). The salt-enriched waters drifted to the seafloor and accumulated in geographical depressions where the brine pools remain stable because of their high density (DasSarma and Arora, 2001). The combination of different extreme physicochemical parameters makes the deep-sea anoxic brine pools one of the most remote, challenging and extreme environments on Earth, while remaining one of the least studied (Antunes et al., 2011). The Red Sea brine pools are extreme in salinity and show a characteristic sharp brine-seawater interface with steep gradients of dissolved O_2_, density, pH, salinity, and temperature (Emery et al., 1969; Ross, 1972; Anschutz and Blanc, 1995). Except for the connected brine pools Atlantis II, Chain, and Discovery Deep (Backer and Schoell, 1972; Faber et al., 1998), environmental conditions vary drastically between the pools, e.g., temperatures range from 22.6°C (Oceanographer) to 68.2°C (Atlantis II) and the NaCl concentration vary from 2.6 M (Suakin) to 5.6 M (Discovery) (Antunes et al., 2011). While the brine pools were detected more than 65 years ago by the Swedish RV Albatross expedition (1947–1948) (Bruneau et al., 1953), microbiological analysis did not start until the late 1960's. The first sampling led to the assumption that under the harsh environmental conditions of the brines life is not possible (Watson and Waterbury, 1969). The search for life in those extreme habitats continuously intensified after the high scientific and economic potential of halophilic organisms became evident (Karan et al., 2012). Since 2010, several sampling expeditions to the Red Sea brine pools have provided a large amount of genomic data, which are collected and annotated at KAUST within the recently described Integrated Data Warehouse of Microbial Genomes (INDIGO) data ware house (Alam et al., 2013). Data stored in INDIGO will stepwise become publicly available.

Analysis and management of next generation whole genome sequencing (NGS) data utilizes comprehensive package of software applications for assembly of sequence reads, mapping to reference genome, variants/SNP calling and annotation, transcript assembly/quantification, and identification of sRNA (Horner et al., 2010; Garber et al., 2011; Pabinger et al., 2014), yet further improvements are required (Dolled-Filhart et al., 2013). Large-scale annotation of DNA sequences with a low homology to genes of experimentally verified function may be flawed and hence represents a major drawback for biomining. The homology-based annotation faces one intrinsic issue: annotation reliability and protein diversity are reciprocal. The situation is complicated by error propagation. The function of the encoded protein was validated experimentally only for a small and continuously diminishing fraction of the gene sequences available. Initially, functions of novel genes were annotated based on gene sequences with experimentally verified function. Based on these data more genes were annotated and so on. While in this chain two proteins are always highly similar, the last annotated gene and the experimentally verified source may possess distinct sequences and functions. In comparison to genomic sequencing, experimental characterization of Single Amplified Genome (SAG) gene products requires gene synthesis, expression, purification as well as functional characterization and therefore is by several orders of magnitude more time consuming. Hence, false positive results from flawed annotation are much more problematic than false negative (due to non-complete annotation) when genomic data are searched for a desired function. This is particularly true for genes from extremophilic organisms, which require slow growing expression systems. Here we present a strategy to minimize false positive identification of the gene product's function. The Profile and Pattern Matching (PPM) algorithm describe below collates complementary information available from (a) InterPro-derived Gene Ontology (GO) terms (Ashburner et al., 2000), which connect an enzyme's function to amino acid sequence profiles and (b) annotated PROSITE IDs (Sigrist et al., 2013), which are linked to an amino acid consensus pattern. This PPM algorithm was tested on 15 protein families of scientific or commercial interest. The strict PPM algorithm initially extracted the most reliably annotated genes, which in this example represent about 1.5% of the genes in the database. Subsequent removal of incomplete genes followed by PPM selection lead to further condensation of gene hits (0.1% of genes in database). A final ranking extracted 11 genes as most likely candidates to code for one of the Protein of Interest (POI) functions.

## Materials and methods

### Sample collection

All samples were collected during leg 2 of the RV *Aegaeo* WHOI, AUC—KAUST Red Sea Cruise in October/November 2011. Samples were taken at different depths and locations in the Red Sea, in and outside the brine pools as well as from sediments. For all brine pools, samples were taken in the brine itself, the sediment and at different depths of the brine seawater interphase (Eder et al., 2001). In total 46 casts were done containing 7030 L of water, as well as seven sediment samples. The collected liquid samples were immediately filtered using a TFF (tangential flow filtration) system, concentrated and immediately afterwards stored at −80°C. During the sampling, different chemical parameters including salinity (conductivity) and temperature were measured. The five brine pools sampled were Kebrit Deep, Nereus Deep, Atlantis II Deep, Discovery Deep, and Erba Deep (Backer and Schoell, 1972; Searle and Ross, 1975; Karbe, 1987; Hartmann et al., 1998).

### Single amplified genome generation

For the production of SAG from single cells, the “SCGC SAG generation service” (cat. no. S-101) at the “BIGELOW Laboratory single cell genomics center,” which is part of the Bigelow Laboratory for Ocean Sciences in Boothbay Harbor, Lincoln County, Maine, United States, was used. The service includes initial sample evaluation for FACS suitability, individual cell separation into wells of a 384-well plate, cell lysis, and single cell multiple displacement amplification (MDA).

### Whole genome sequencing and assembly

The whole genome sequencing was performed at the “BIGELOW Laboratory single cell genomics center” using the “Prokaryote SAG whole genome sequencing” service (cat. no. S-014). The service includes sequencing library preparation, genomic sequencing, *de novo* assembly, and assembly quality control. Service products include contig fasta files and assembly statistics. Assemblies of the single-cell amplified genomes (SAGs) were generated using a pipeline that employs a choice of assemblers designed for single-cell sequencing data including VelvetSC (Chitsaz et al., 2011), SPAdes (Bankevich et al., 2012), and IDBA-UD (Peng et al., 2012), along with several pre- and post-assembly data quality checks using Trimmomatic (Lohse et al., 2012). IDBA-UD was benchmarked as the overall best assembler for our SAGs as is it did reconstruct longer contigs with higher accuracy to the reference genome of *Nitrosopumilus maritimus* SCM1 (Könneke et al., 2005).

### Dataset

The data used in this work consisted of 87 SAGs covering 16 different taxonomic groups, sampled in 11 different environments. A total of 26,626 contigs covering 111,269 ORFs and containing 79.8 Mbp genomic information (Table 1) were analyzed.

**Table 1:** **Two example and summary (*italic*) of the SAG data in INDIGO used for this work**.

**Organism**	**Habitat °C; % salt)**	**Contigs (number)**	**Contigs (min. bp)**	**Genome (Mbp)**	**N50 (kbp)**	**N90 (kbp)**	**ORFs**
SAR86 clade	25.0; 4.0	86	312	0.74	83	6.9	853
MSBL1	54.0; 15.2	849	200	2.20	15	0.7	3293
*87 SAGs, 16*	*11 types*	*26,626*	*200 – 317*	*79.8*	*1,4 – 1,200*	*0.4 – 13.9*	*111,269*
*taxonomic groups*	*23.4 – 63.0; 4 – 26*	*(16 – 849)*		*(0.1 – 2.2)*			*(242 – 3,293)*

#### Annotation of the dataset

**Figure 1:**
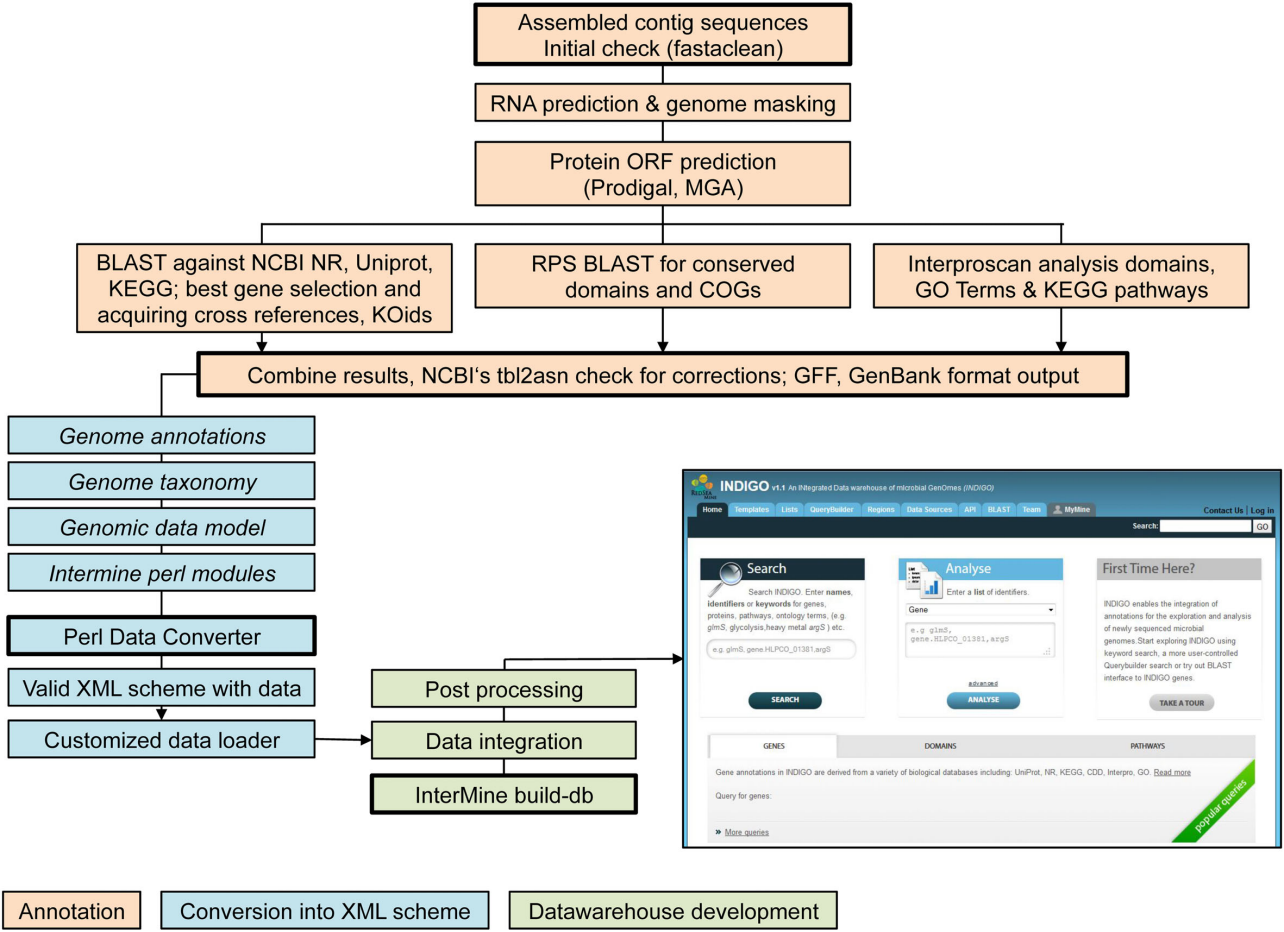
**Workflow of data integration into the INDIGO warehouse starting from assembled contig sequences**.

The assembled contig sequences were integrated into the INDIGO data warehouse (Alam et al., 2013) for microbial genomes. INDIGO is a dynamic system using the InterMine framework (Smith et al., 2012), one of the highest benchmarked data warehouses (Triplet and Butler, 2013). INDIGO allows Automatic Annotation of Microbial Genomes (AAMG), extensive query building for annotation integration, creation of customized feature/attribute/entity lists and enrichment analysis for GO concepts, which are crucial steps of the following analysis. Using INDIGO the assembled contig sequences were (i) annotated, (ii) converted into an XML schema, and (iii) implemented into the data warehouse. Figure 1 gives an overview of the workflow (Alam et al., 2013). Assignments of GO-terms are largely independent from PROSITE IDs. GO-terms emerge from domain associations provided by InterPro (Quevillon et al., 2005) (one of several domain resources may be PROSITE). PROSITE consensus patterns are predicted by the PS_Scan (De Castro et al., 2006) tool.

***Automatic annotation of microbial genomes (AAMG) pipeline***. Functional annotation of archaeal or bacterial genomes is available via the INDIGO website interface (http://www.cbrc.kaust.edu.sa/indigo/mymine.do?subtab=aamg). Completed genome annotations may be included into the INDIGO database. This enables application of the scripts presented in this work for any novel genetic data.

### Phylogenetic analysis

The evolutionary history was inferred using the Neighbor-Joining method (Saitou and Nei, 1987). All illustrated trees are drawn to scale, with branch lengths in the same units as those of the evolutionary distances used to infer the phylogenetic tree. The evolutionary distances were computed using the Poisson correction method (Zuckerkandl and Pauling, 1965) and are in the units of the number of amino acid substitutions per site. All positions containing gaps and missing data were eliminated. Evolutionary analyses were conducted in MEGA6 (Tamura et al., 2013).

### PPM methodology

The PPM algorithm was automated by including two new scripts into INDIGO, which are publicly available from the homepage.

#### AutoTECNo: automated translation of E.C. numbers

The E.C. No. translator (AutoTECNo) automatically converts a list of given enzyme commission (E.C.) numbers into GO-terms (Kanehisa and Goto, 2000) as well as PROSITE IDs, using open source PROSITE files (Sigrist et al., 2002). Preliminary, transferred and deleted E.C. numbers are ignored. The AutoTECNo provides two XML scripts for the independent profile and pattern search via INDIGO. AutoTECNo is available at the following website: http://www.cbrc.kaust.edu.sa/ppma/ec2gops.html.

#### PPM processor: automated extraction and ranking of the most reliable hits

The PPM Processor requires one or more tab separated spreadsheets (.tsv) of the independent profile analysis (via GO-terms) and/or pattern analysis (via PROSITE IDs) as input file. The processor generates sets of genes according to their profile and pattern distribution. The resulting list is ranked regarding to the amount of profile and pattern combinations. The PPM processor is available at the following website: http://www.cbrc.kaust.edu.sa/ppma/indigoTbl2PSgoSets.html.

#### The PPM workflow, starting from a non annotated genome

First, an assembled genome is annotated using the AAMG pipeline as part of the INDIGO data warehouse. Second the E.C. number based list of POI (list) is translated into profile and pattern values (GO-terms and PROSITE IDs) by using AutoTECNo. The resulting XML lists (of pattern and profile values) are separately imported into the INDIGO data warehouse to analyze any listed genome at the following URL: http://www.cbrc.kaust.edu.sa/indigo/importQueries.do?query_builder=yes. The two resulting tab separated spreadsheets can be uploaded into the PPM processor to generate three PPM sets of genes: (i) profile set, (ii) pattern set, and (iii) profile and pattern set.

## Results

### PPM: profile and pattern matching for function identification

Analysis of the huge amount of data resulting from next generation whole genome sequencing (NGS) requires modern bioinformatic tools. Comparisons of annotation pipelines reveal a surprising level of uncertainty in gene annotation. Annotations of the same genome (strain TY2482) of the enterohemorrhagic diarrhea causing shiga-toxin-producing *E. coli* O104 (Rohde et al., 2011) by several groups allowed a comparison of the three main annotation pipelines: Broad, BG7, and RAST. Compared 5164 coding sequences (CDS) of to the Broad annotation the BG7 annotation resulted in 5210 CDS with 163 (3.1%) false negatives and 271 (5.2%) false positives, and RAST annotation gave in 5446 CDS with 116 (2.1%) false negatives and 321 (5.9%) false positives (Alam et al., 2013). The AAMG based annotation stored in INDIGO, which is used for this article, gave results similar to those of the Broad institute. Annotation of the *E. coli* K12 strain W3110 by INDIGO resulted in 4340 CDS (NCBI 4337), with 236 (5.4%) false positives and 235 (5.4%) false negatives in comparison to the NCBI annotation. These examples illustrate, that state-of-the-art annotation still yields about 5.5% false positives for strains of the standard organisms *E. coli* and a significantly higher rate of false positives may be expected for novel genomes. While this might not impact *in silico* analysis e.g., for identification of pathways, a substantial amount of false positives can lead to costly failures in experimental bioprospecting campaigns.

Among the descriptors INDIGO annotation associates with genes, two are particularly suited to evaluate the correct assignment of an enzymatic function to a gene product: (i) the GO-term and (ii) the PROSITE ID. The GO project describes genes (gene products) using terms from three structured vocabularies: biological process, cellular component and molecular function. Correspondingly, a list of GO-terms associated with a gene can be seen as the gene's profile. A PROSITE ID relates to a single consensus pattern as “amino acid sequence signature” to characterize protein function. Genes from INDIGO with matching function description of GO-term and PROSITE ID(s) should represent a subset of genes with highly reliable annotation. To extract such genes based on an input list of E.C. numbers of interest, we developed a protein PPM algorithm.

#### From proteins of interest to bioinformatics descriptors

Initially, we established a set of proteins, which potentially are of scientific and/or commercial interest. Protein classes selected include a variety of hydrolases, ene reductases, dehydrogenases, and carbonic anhydrases (CAs) as well as a range of metalloproteins, porines and potentially new aminoacyl tRNA synthetases. The selected 15 protein families of interest (POI families) are summarized in Table 2. Bioinformatic matching of the POIs vs. the INDIGO database requires a translation of the POI list into terms of the selected descriptors (GO-terms and PROSITE ID). For enzymes, E.C. numbers can be associated with the enzyme family name as well as GO-terms and PROSITE ID and therefore can be used to interconvert these terms. The POI list was translated into the E.C. numbers using BRENDA (Braunschweig Enzyme Database) (Schomburg et al., 2013). Of the resulting 2577 E.C. numbers (Table S1) 434 were non-redundant. Removal of preliminary/transferred and deleted E.C. numbers provided a final list of 265 E.C. numbers (Table S2). The list of E.C. numbers was converted into profiles (GO-terms) and pattern (PROSITE IDs). For gene expression products without enzymatic function like aquaporins and pyltRNA, the respective GO-terms and PROSITE IDs were added manually. The resulting protein profile filter consist of 171 non-redundant GO-terms (BRENDA) (Table S3). The independent pattern filter consisted of 52 non-redundant PROSITE consensus pattern (Sigrist et al., 2013). Three consensus patterns (PS00198, PS00455, PS00143) were removed because of their low specificity (consensus pattern specificity can be derived from the information available at PROSITE web page: http://prosite.expasy.org), resulting in a final pattern list of 49 consensus pattern (Table S4).

**Table 2:** **List of proteins of interest (POIs), which were selected for this study**.

**No**	**POI group**	**Description**	**Interest**
1	Alcohol DH	Interconversion of aldehydes/ketones and alcohols	Biocatalytic synthesis of chiral intermediates
2	Formate DH	Conversion of CO_2_ into format	Biological carbon capture
3	Formaldehyde DH	Interconversion of formaldehyde and formate	Biological carbon capture, methanol conversion
4	Carbon monoxide DH	Interconversion of CO and CO_2_	Biological carbon capture, metalloenzyme structures
5	Ene reductase	Stereoselective reduction of alkenes	Biocatalytic synthesis of chiral intermediates
6	Protease	Hydrolysis of peptide bonds	Detergents, food, and leather processing
7	Terpene synthase	Synthesis of basic, (mulit-)cyclic terpene structures	Biocatalytic synthesis of complex intermediates
8	Nitrogenase	Fixation of nitrogen from air	metalloenzyme structure and function
9	Lipase	Hydrolysis of triglyceride esters	Detergents, biodiesel synthesis
10	Carbonic anhydrase	Interconversion of CO_2_ and Bicarbonate	Biological carbon capture, metalloenzyme structures
11	Acetylene hydratase	Synthesis of aldehydes from acetylene	Biocatalytic synthesis intermediates
12	Acetyl-CoA synthetase	Activation of acetate for further conversion	Biological carbon capture metabolism
13	pylRS	Aminoacyl tRNA synthetase, acting on pyrrolysine	Synthetic biology, expanding the genetic code
14	pyltRNA	tRNA coding for pyrrolysine	Synthetic biology, expanding the genetic code
15	Aquaporin	Integral membrane proteins controlling osmotic pressure	Water desalination membranes

***AutoTECNo: automated translation of E.C. numbers***. The web-based AutoTECNo script simplifies conversion of POI classes into the two bioinformatic PPM descriptors described above. A user may enter one or more distinct or flexible E.C. numbers, which are automatically converted into GO-terms and PROSITE IDs. A numeric value is required for the first three digits of flexible E.C. numbers (e.g., 1.1.1.^*^). AutoTECNo automatically ignores preliminary, transferred and deleted E.C. numbers. The AutoTECNo output provides two XML scripts, one for each of the independent profile and pattern search, which can be imported directly into the INDIGO data warehouse by using the direct links on the output page.

#### The PPM (profile and pattern matching) algorithm

**Figure 2:**
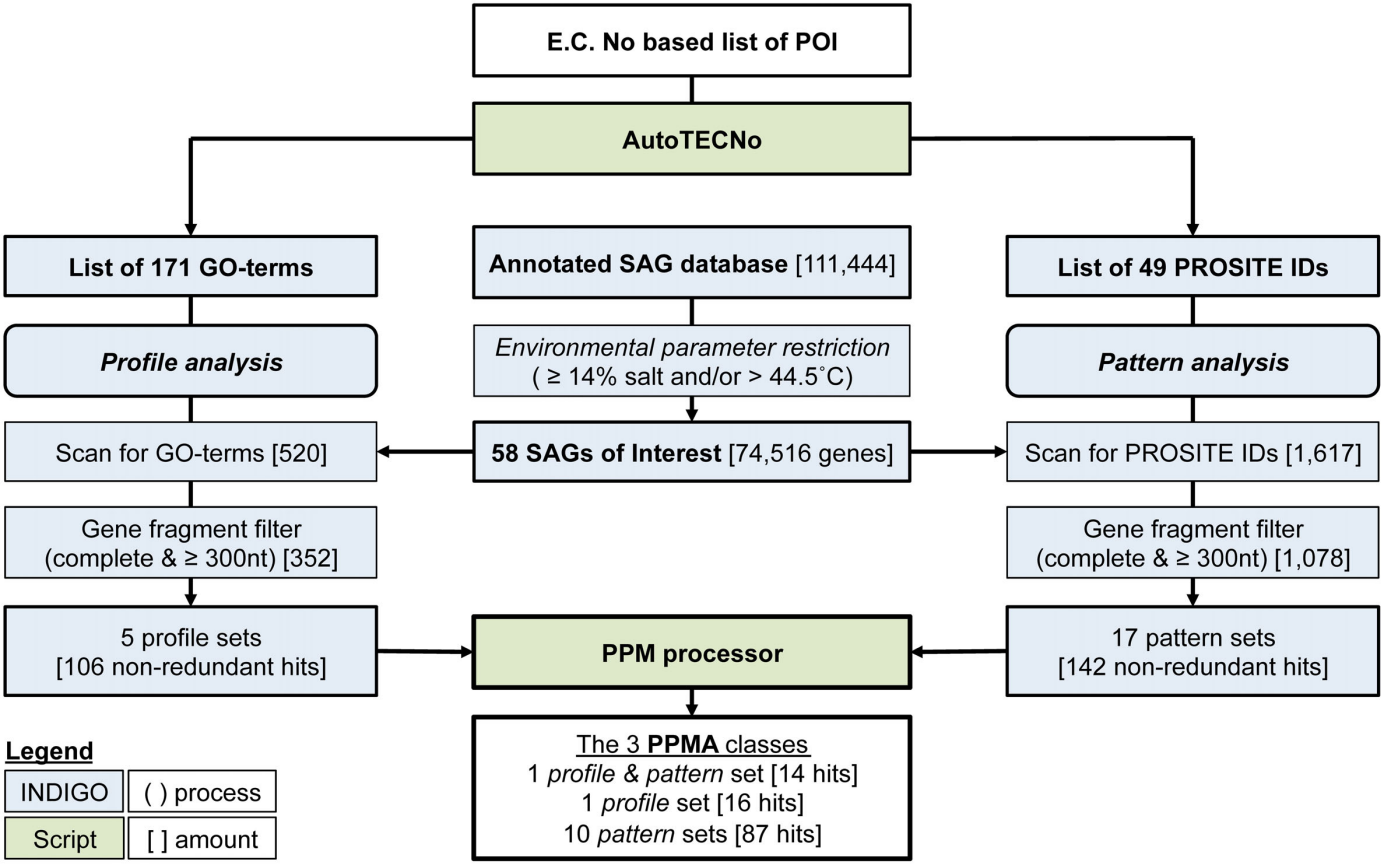
**Flowchart illustrating the PPM (profile pattern matching) algorithm, starting from an E.C**. Number based proteins of interest (POI) list and a selected database subset, which may also be uploaded externally. Numbers refer to the example published here. Square brackets indicate number of genes at each step during the example analysis, specific restriction filters are described in normal brackets. The complete PPM algorithm is available at the INDIGO webpage including the scripts AutoTECNo and PPM Processor.

The PPM algorithm retrieves those POIs from a database, which are most likely to be annotated correctly. Initially, the GO-term list (profile) and the consensus pattern list (coded by the PROSITE IDs) are matched independently onto the dataset of interest. From each of the resulting subset of genomic data, gene fragments commonly present in SAGs or metagenomic data a gene fragment filter eliminates (i) genes with less than 300 nucleotides (to sustain a minimal length required for functionality) and (ii) genes that are not annotated as complete (indicating that a 3' or 5' part of the gene is missing). In a last step, both filtered lists are transferred to the PPM processor (see below), which arranges all hits into sets of genes having the same combination of identifiers (GO-terms and/or Prosite IDs). Three classes of sets are listed: (i) the profile sets, containing genes with one or more GO-term describing the respective POI, (ii) the pattern sets, containing genes with one or more PROSITE ID of the respective POI and (iii) the profile and pattern set, consisting of genes with at least one GO-term and PROSIT ID of the POI. The annotation of genes is ranked as more reliable with increasing numbers of associated identifiers. The complete PPM algorithm is illustrated in Figure 2.

Identification of the most reliably annotated genes in INDIGO that match our POI served as test-case for the PPM. The genetic database search was restricted to certain brine pool SAGs based on environmental parameters of the sampling locations (salinity ≥ 14% and/or a temperature >44.5°C). The habitats selected were set to reflect the upper part of moderate halophilic conditions (5–20% salt) as well as extreme halophilic conditions (20–30% salt) (Ollivier et al., 1994) and/or thermophilic conditions [45–80°C (Madigan et al., 2003)]. The sample subset comprises 58 SAGs from three different brine pools (Atlantis II deep, Discovery, and Kebrit), covering six different environmental conditions. These SAGs contain a total of 73,688 ORFs coding for 74,516 genes. The ORFs were assembled out of 21,519 contigs into genomes of a combined size of 48.2 mega base pairs (Table 3).

**Table 3:** **Bacterial (*italic*) and archeal SAGs from thermophilic and hypersalinic sampling regions selected for this study**.

**SAGs**	**Taxonomy**	**Genome (Mbp)**	**Contigs**	**ORFs**	**Salinity [%(w/v)]**	**T (°C)**	**Location**
1	*Desulfo-bacterales*	1.2	401	1658	9.4	47	Atlantis II
13	MSBL1	8.8	4262	14,159	16.8	63	
10	MBGE	9.5	4809	14,809			
3	MSBL1	2.8	1019	4114	15.2	54	
3	SA2 cluster	0.9	386	1367			
1	*Candidate division*	0.4	176	598			
2	MSBL1	1.2	321	1716	14	32	Discovery
17	MSBL1	17.9	7462	27,110	26.2	44.8	
5	MSBL1	3.2	1647	4989	26	23.4	Kebrit
3	*UMSBL6*	2.3	1036	3168			
58	6 taxon. groups	48.2	21,519	73,688	6 habitats		3 pools

As described above, the POI list was transformed into a protein profile filter consisting of 171 non-redundant GO-terms (BRENDA) and an independent pattern filter of 49 PROSITE IDs (Sigrist et al., 2013). Profile matching of the 74,516 preselected genes with the 171 GO-terms resulted in 520 hits, which were further reduced by the gene fragment filter to 352 (Table 4). Elimination of duplicates (genes associated to multiple GO-term or PROSITE ID occur multiple times in the output) yielded 106 non-redundant hits, which could then be grouped into five different profile sets, based on the gene-associated GO-terms. The five profile sets contain six different GO-terms, four profiles with only one GO-term and one profile with two GO-terms (Table 5). Categorizing the 106 genes into five profile sets clarifies what functions and functional diversity can be expected from the hits.

**Table 4:** **Stepwise overview of the conversion the 15 selected enzyme groups into non-redundant GO terms and PROSITE ID**.

**No**	**POI-group**	**E.C.-No**.			**GO-terms**			**PROSITE IDs**		
		**T**	**NR**	**S**	**T**	**NR**	**Hits**	**T**	**NR**	**Hits**
1	Alcohol DH	101	32	25	20	20	0	12	8	2–6
2	Formate DH	29	6	6	4	4	1	7	6	6
3	Formaldehyde DH	23	9	4	3	2	0	0	0	0
4	Carbon monoxide DH	19	4	4	4	4	1	1	0	0
5	Ene reductase	1162	107	65	61	61	4	9	1	1–5
6	Protease	741	217	111	39	39	0	45	20	8
7	Terpene synthase	35	023	17	9	9	0	0	0	0
8	Nitrogenase	18	4	2	2	2	0	4	4	4
9	Lipase	380	26	25	24	24	0	9	6	0
10	Carbonic anhydrase	58	1	1	1	1	0	3	3	0
11	Acetylene hydratase	2	1	1	1	1	0	0	0	0
12	Acetyl-CoA synthetase	8	3	3	3	2	0	1	0	0
13	Aquaporin	0	0	0	2	2	0	1	1	0
Total		2576	433	264	173	171	6	92	49	25

T, Total in class; NR, non-redundant ones; S, selected for this study.

**Table 5:** **Enzyme hits identified using the PPM algorithm and the respective PPM descriptors**.

**PPM class**	**Profile (GO-term) or pattern (PROSITE IDs)**			**Hits**	**PPM set**	**Enzyme hit**
Profile	GO:0008839			3	–	
	GO:0009326			25	–	
	GO:0018492			17	–	
	GO:0043115			31	–	
	GO:0004665	GO:0008977		16	Pro 1	Prephenate DH [1.3.1.13]
Pattern	PS00059			15	–	
	PS00061			3	–	
	PS00136			3	–	
	PS00137			1	–	
	PS00138			2	–	
	PS00141			11	–	
	PS00501			6	–	
	PS00060	PS00913		4	Pat 1	Fe—ADH [1.1.1.1]
	PS00062	PS00063	PS00798	19	Pat 2	dkgA [1.1.1.274]
	PS00065	PS00670	PS00671	27	Pat 3	Glyoxylate red. [1.1.1.26]
	PS00381	PS00382		2	Pat 4	Clp protease [3.4.21.92]
	PS00490	PS00551	PS00932	11	Pat 5	Molybdopt. OR [e.g., 1.2.2.1]
	PS00090	PS00699		10	Pat 6	Nitrogenase [1.18.6.1]
	PS00692	PS00746		7	Pat 7	
	PS00136	PS00137		2	Pat 8	Subtilisin [3.4.21.*]
	PS00136	PS00138		4	Pat 9	
	PS00137	PS00138		1	Pat 10	
Profile and Pattern	GO:0008839	PS01298		14	PP 1	DHPR [1.3.1.26]

The independent pattern filter was applied according to the same scheme. Screening all 58 SAGs against the 49 PROSITE IDs resulted in 1617 hits. Applying the gene reliability filter reduced this number to 1078 hits, which could be further condensed to 142 non-redundant hits. These 142 genes fall into 17 pattern sets containing 25 different PROSITE IDs.

Since the presence of several GO terms, PROSITE IDs or a combination of both indicates a more reliable gene annotation, we used the PPM processor to identify genes which are associated with multiple descriptors. The list (Table 5) contains three sub-sets: (i) the profile sets (one set of 16 hits), (ii) the pattern sets (10 sets containing 87 hits), and (iii) the profile and pattern sets (one set of 14 hits). Only the profile and pattern set contains genes, which were found independently by both, PPM. In other words, when the INDIGO subset of 74,516 genes is screened for the 434 non-redundant E.C. numbers, only 14 genes have a matching GO-term and PROSITE ID. All 14 hits belong to the same E.C. number (1.3.1.26, dihydrodipicolinate reductase, DHPR). Since some profile or pattern sets stand for the same enzyme type the total amount of 117 most reliably annotated genes that were identified by the PPM algorithm fall under only nine different enzyme families: prephenate DH (1.3.1.13), iron containing ADH (1.1.1.1), dkgA (1.1.1.274), glyoxylate reductase (1.1.1.26), Clp protease (3.4.21.92), molybdopterin oxidoreductase (e.g., 1.2.2.1), nitrogenase (1.18.6.1), subtilisin (3.4.21.^*^), and DHPR (1.3.1.26). The relatively small number of highly reliable hits is helpful for an experimental scientist, who is aiming to characterize novel gene expression products. A reduction of 111,444 potential expression targets to only 117 provides the necessary experimental focus (see below).

***Semi-automatic, XML based PPM algorithm***. The PPM algorithm was integrated into the INDIGO web page via a XML script. The semi-automated work flow requires three steps: (i) conversion of the POI list into GO-terms and PROSITE IDS with AutoTECNo, (ii) individual profile as well as pattern matching via a query in INDIGO and (iii) extraction and ranking of the most reliable result in pattern, profile and profile and pattern by the PPM processor. This process requires two input files: (i) an assembled genome, which can be annotated using the AAMG pipeline and (ii) an E.C. number based POI list. The POI list can directly be copied into the AutoTECNo input mask. After submitting the E.C. number list, AutoTECNo will generate a list of all E.C. numbers and the associated GO-terms and PROSITE IDs. At the bottom of the output mask, three links are provided: “GO xml,” “Prosite xml,” and “INDIGO datawarehouse.” Clicking either of the first two links will open a window, which provides.xml-formatted files (for either GO-terms or PROSITE IDs). These files can be edited and used separately to build INDIGO queries. In such a query, INDIGO is used to match each of the two.xml lists against the selected genomes. Clicking on the “INDIGO datawarehouse” link opens the INDIGO XML input mask, which can be used to initiate a query by pasting the.xml script from AutoTECNo. A graphical overview of the query will be shown and further customization can be done (pre-set columns should not be deleted). At this stage, both, profile (GO-term) and pattern (Prosite ID) filters can be applied individually in connection with the optional gene fragment filter. Hits will be organized in a table summarizing all information available in INDIGO. The table still may contain duplicates, since one gene can be found under several GO-terms and/or PROSITE IDs. The results-table can be downloaded as “Spreadsheet (tab separated values)” (.tsv file) for import into the PPM processor. The PPM processor output provides a list of non-redundant genes, grouped into subsets of the three classes of hits (profile sets, pattern sets and profile and pattern sets) as well as ranked based on the amount of associated patterns and profiles. A link back to INDIGO allows listing of the obtained hits for a detailed analysis.

### Manual hit selection from the PPM processor output

Grouping of genes into PPM classes and sets immediately highlights expected functional similarities of gene expression products. PPM sets of patterns and/or profiles, which are characteristic for the same protein, can be condensed further into one meta-set. For example pattern sets with combinations of PS00136 and PS00137, PS00136, and PS00138 or PS00137 and PS00138 are all indicative of subtilase type serine proteases and these pattern sets were condensed into one meta set. In total nine functionally distinct PPM sets remained after manual condensing (Table 5).

**Figure 3:**
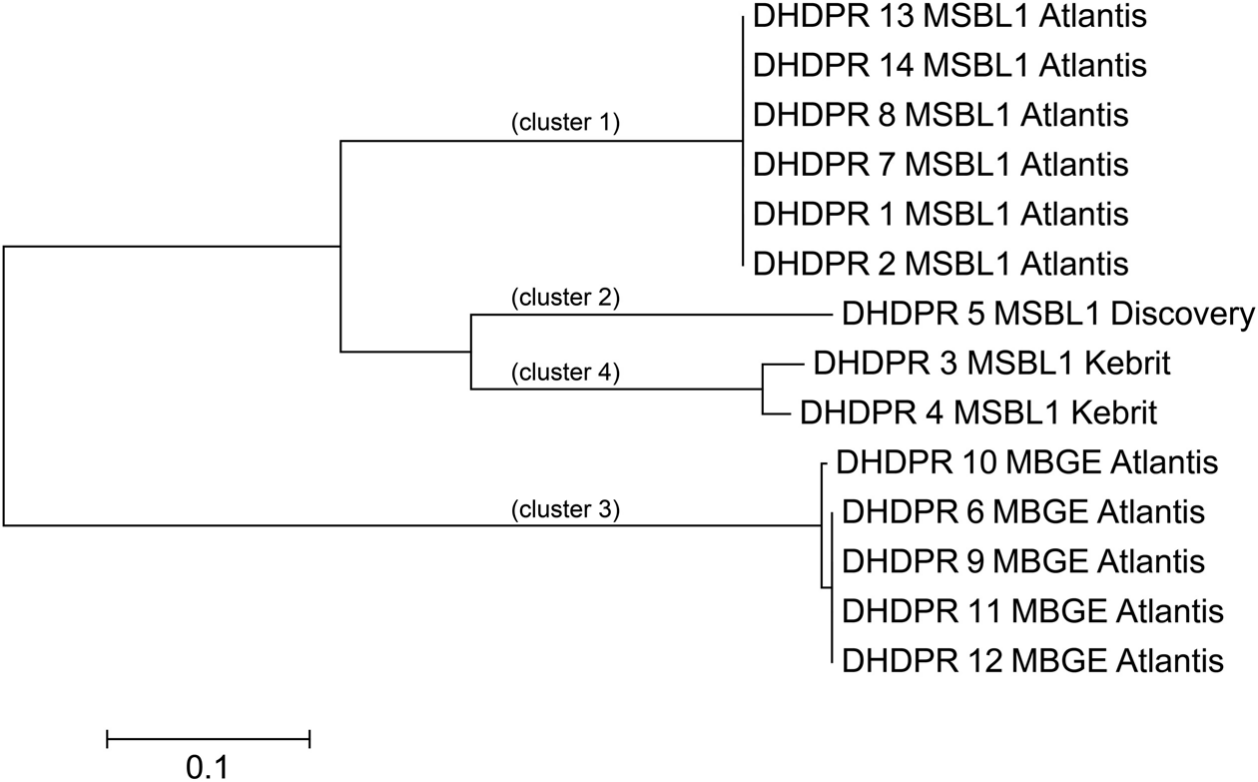
**Phylogenetic tree of the DHPR hits**. To identify isoenzyme classes, every PPM set of hits was clustered into phylogenetic groups. For the 14 Dihydrodipicolinate reductases (DHPR) four closely related phylogenetic clusters were found. Scale bare: 0.1 amino acid substitutions per site.

For experimental characterization, synthesis and expression of 117 genes from halophilic extremophiles still represent an enormous challenge, which mandates identification of those extremophilic proteins as expression targets, which are most typical for each functionality-set. For five of the nine functionally different PPM sets, we were able to pinpoint nine genes representing all three PPM classes (profile, pattern, profile, and pattern) (Table 5). Amino acid based phylogenetic analysis within each PPM set revealed phylogenetic relations and sequence clusters. The sequence representing most of the set-members was selected, e.g., the PP1 DHPR PPM set contains 14 different hit sequences (isoenzymes). Phylogenetic analysis resulted in four clusters of phylogenetic closely related groups (Figure 3). For each of those four clusters the sequence representing most of the members was selected. This was straightforward for three DHPR clusters, since one sequence contained all elements of the others. In the fourth case as well as for cluster of other sets the selection was more complicated, because phylogenetic sequence clusters showed either an equal distribution of mutations in one cluster or an unequal length of sequences. To address this problem an additional protein BLAST (BLASTp) (Johnson et al., 2008) was performed and the sequence with the highest similarity was chosen for the fourth DHPR and the halolysin cluster. In case of no difference in similarity according to BLASTp, the gene product providing more functional side chains was chosen (e.g., for subtilisin) since additional chemical functionality may indicate more diverse enzyme characteristics (e.g., hydrogen bonding, allosteric pockets, metal complexation etc.). Amino acid sequences typically differed in less than 10 positions [amino acid sequence length: 401 (ADH), 348 (2-hydroxyacid DH), 498–565 (halolysin; the 565 amino acid sequence contains all shorter ones), 528 (subtilisin), 435–440 (prephenate DH), 272–285 (four subgroups of DHRPs)].

### Function identification of proteins without existing GO-terms or prosite IDs

The initial search for CAs was not successful. While distinct GO-terms and consensus patterns exist for α- and β-CAs (Table 5), non are available for the other three CA families (γ, δ and ζ). According to Ferry the CS chain A from *Methanosarcina thermophile* (Cam) can be considered the archetype of the γ-CA family, and a distinct, 180 amino acid sequence (no 34–214) is indicative for a γ-CA protein (Smith and Ferry, 2000). An INDIGO internal BLAST of this 180 amino acid motif against all genes yielded 17 potential γ-CAs. Applying the gene fragment filter reduced the candidate pool to six.

As discussed above, an additional pattern matching should increase the reliability of the profile-based protein identification. The analysis of the only two γ-CA class crystal structures reported (Cam from *M. thermophila* Kisker et al., 1996, see also pdb 3OW5 and a CamH homolog from *P. horikoshii* Jeyakanthan et al., 2008) revealed nine amino acids in two peptide sequences of 26 and six amino acids as most relevant for enzyme function (Smith and Ferry, 2000). The resulting two initial consensus patterns are shown below [color code: yellow, metal binding motifs (H_81_, H_117_, H_122_); green, residues directly involved in catalysis (E_62_, N_73_, Q_75_, E_84_); blue, structurally important residues (R_59_, D_61_); not highlighted, residues of no specific function as they appear in the γ-CA sequence].

59 - 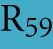
S
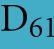

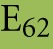
GMPIFVGDRS
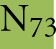
V
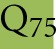
DGVVL
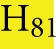
AL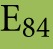 - 84 and

117 - 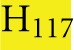
QSQV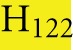 - 122

No hit was found for a strict pattern matching of the six potential γ-CAs. This is not surprising, since it is common for consensus patterns that some functionally important amino acids can be altered within a certain threshold. Alignment of the initial γ-CA consensus pattern with the six γ-CA candidate sequences revealed that the 10 amino acid long stretch from E_62_ to N_73_ was shortened by one amino acid in all six candidates. The resulting structural alteration is unlikely to affect function. Further, the two structurally important residues R_59_ and D_61_ were conserved as well as two out of the three metal binding histidines (H_81_ and H_117_) (Table 6). The third metal binding amino acid H_122_ was replaced by an N in hit number 6, a mutation, which potentially affects function. Further sequence variations involve the replacement of catalytic E_84_ by either D (four cases, potentially not influencing function), or K (two cases, potentially affecting function). The remaining catalytically important residues E_62_, N_73_, and E_75_, which are involved in a hydrogen-bonding network in the *M. thermophile* protein, are highly variable among the six candidates sequences. Assuming that some of these candidates are CAs because of profile and pattern similarity to the *M. thermophile* archetype enzyme we concluded that E_62_ is not generally important for the function of this enzyme type and that N_73_ and E_75_ can be replaced by the hydrogen bonding amino acids C or K, respectively. Correspondingly, we suggest the following two consensus patterns for γ-CAs:

**Table 6:**
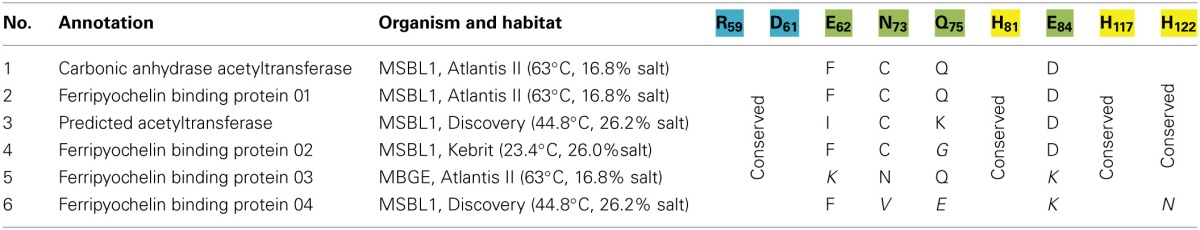
**Alignment of functional important residues the γ-CA chain A from *Methanosarcina thermophile* with γ-CA candidates**.

R-x-D-x(10,11)-[NC]-x-[QK]-x(5)-H-x(2)-[ED] and

H-x(3)-H

Application of the PPM algorithm using the 180 amino acid profile stretch identified from pdb 3OW5 and the new consensus patterns delivered three γ-CAs candidates. Because of high sequence similarity in two out of the three sequences, the sequences of gene 2 (annotated as ferripyochelin binding protein 01) from Atlantis II deep and gene 3 (annotated as predicted acetyltransferase) from discovery deep (Table 6) were selected as best candidates for experimental studies of γ-CAs [CA_A (Atlantis II deep) and CA_D (Discovery deep) in Table 7].

**Table 7:** **Hits identified as reliable using the PPM algorithm**.

**Gen**	**Enzyme [organism]**	**PPM ident**.	**Closest related protein (sequence identity) [organism]**
AF_D	ADH [MSBL1]	Pat 1	ADH, iron-containing (60%) [*Thermotoga neapolitana* DSM 4359]
HD_K	2-Hydroxyacid DH [USMBL6]	Pat 3	NAD-binding 2-hydroxyacid DH (63%) [*Petrotoga mobilis* SJ95]
HP_D	Halolysin [MSBL1]	Pat 8-10	Peptidase S8 (53%) [*Haladaptatus paucihalophilus*]
SP_A	Subtilisin [MBGE]		Peptidase S8 (50%) [*Bacillus* sp. SG-1]
PD_A	Prephenate DH [MBGE]	Pro 1	Prephenate dehydrogenase (48) [*Methanobacterium formicicum*]
DR_A1	DHPR [MSBL1]	PP 1	DHPR (56%) [*Methanobacterium* sp. SWAN-1]
DR_A2	DHPR [MBGE]		DHPR (57%) [*Methanocaldococcus vulcanius* M7]
DR_D	DHPR [MSBL1]		DHPR (54%) [*Methanobacterium* sp. SWAN-1]
DR_K	DHPR [MSBL1]		DHPR (53%) [*Methanobacterium* sp. SWAN-1]
CA_A	γ-CA [MSBL1]	Manual PPM	Ferripyochelin binding protein (fbp, 43%) [*Thermosediminibacter oceani* DSM 16646]
CA_D	γ-CA [MSBL1]		Hypothetical protein (53%) [*Corynebacterium pyruviciproducens*]

Last letter of gene name indicates habitat: D, Discovery; A, Atlantis II; K, Kebrit.

## Discussion

Proteins, which are suitable for the harsh conditions of many biotechnological applications can be obtained through protein engineering, discovery and mining of novel extremophilic genomes or a combination of both. The major challenge in mining genomic data from extreme environments is, that, with increasing extremeness of the habitat, the possibility of culturing the organism thriving under these conditions shrinks substantially (Alain and Querellou, 2009). However, SAGs can provide genomic data from uncultured organism. We believe that improving the quality of SAGs assemblies (higher sequence coverage, longer contigs, and advanced annotation programs) should enable us to utilize SAGs as a rich source for discovery of extremophilic enzymes of scientific interest and commercial value. However, annotation reliability is lowered for both, extremophilic genomes (for which commonly no close relative is known) and SAGs (which may suffer from gaps, incomplete genes, or generally sequencing data of lower quality) and therefore a highly reliable algorithm for identification of genes of interest from extremophilic SAG databases is mandatory before entering labor-intensive expression and characterization of these genes.

### Problems of single profile or pattern analysis and the PPM algorithm

Consensus patterns show a good reliability, yet a considerable amount of hits identified via PROSITE ID are false positives (has the motif but not the function), false negatives (has the function but not the motif), unknown (has the motif but no verified function), or partial hits (has the function but only parts of the motif) (Sigrist et al., 2002). Table 8 combines examples illustrating the reliability for consensus pattern based annotation of enzyme function. Reliability may be as low as 55% false positives (PS00136) or 90% false negatives (PS00065). A further problem of pattern-based annotation is the low flexibility because of the short pattern lengths (about 10–20 amino acids Sigrist et al., 2002), typically covering only 1.9–7.9% of the total protein length. Due to the short length of the consensus pattern, a higher reliability requires reducing the permissible flexibility. In the CAs example above, three consensus patterns were available with high reliability (Table 8). Hence we expected to identify several CAs through pattern matching. Yet, no CA was found in the entire database since the rigidity of these consensus patterns prevented identification of novel enzymes with the same function. Finally, a consensus pattern may not be specific for a specific function, e.g., NADH or ATP binding motifs typically are associated with consensus patterns, which occur in several enzyme families. Table 7 illustrates this issue. Four PROSITE IDs are related to both, either alcohol dehydrogenase or ene reductase function. Identifying combinations of patterns can circumvent these problems and increase reliability. According to the PROSITE web page, one of the strongest pattern combinations is PS00136–PS00138. If a protein includes at least two of the three active site signatures, the probability of it showing a protease activity is assumed to be 100%.

**Table 8:** **Reliability of consensus patterns found in chosen hits as well as for carbonic anhydrases**.

**PROSITE ID**	**Description**	**Hits**	**TP (%)**	**FP (%)**	**FN**	**FN (%)**
PS00059	Zinc-containing AD signature	491	97.4	2.6	40	8.1
PS00061	Short-chain DHR family signature	720	82.5	17.5	192	26.7
PS00065	NAD-binding 2-hydroxyacid DH signature	235	77.4	22.6	210	89.4
PS00136	Subtilase family, aspartic acid active site	328	45.1	54.9	90	27.4
PS00137	Subtilase family, histidine active site	200	92.5	7.5	56	28.0
PS00138	Subtilase family, serine active site	261	88.1	11.9	29	11.1
PS00671	NAD-binding 2-hydroxyacid DH signature 3	319	99.7	0.3	66	20.7
PS00913	Iron-containing ADH signature 1	42	81.0	19.0	26	61.9
PS01298	DHPR signature	541	100.0	0.0	19	3.5
PS00162	A-CA signature	64	100.0	0.0	32	50.0
PS00704	Prokaryotic-type CA signature 1	22	95.5	4.5	10	45.5
PS00705	Prokaryotic-type CA signature 2	25	100.0	0.0	5	20.0

TP, True positive; FP, False positive; FN, False negative (Sigrist et al., 2002).

Ontologies are widely used for functional annotation (Radivojac et al., 2013). Gene ontologies are commonly expressed by GO-terms. The source for GO-terms in the UniProt Gene Ontology Annotation database falls into three categories: (i) the smallest but most reliable category, experimental annotations, (ii) curated non-experimental annotations and last electronic annotations, (iii) with less reliability. Over 98% of the repository of the UniProt Gene Ontology Annotation database is inferred in silico without curator oversight (Škunca et al., 2012). GO-terms are highly flexible, which is reflected in the gene's sequence length associated with it, e.g., annotation of GO-terms in this study covered 1.9–100% of the total gene. The particular sources used for GO-term identification leads to this large range. GO-terms based on consensus pattern naturally are reflected by a short associated sequence length (e.g., the 1.9% lower limit in this study). GO-terms determined by different methods (e.g., Hamap, TIGRfam, PIRSF) can take up to 100% of the sequence into consideration. In this analysis GO-terms association to ORFs was in average based on about 65% of the total sequence length. Recent studies could show that electronic annotations are more reliable than generally believed and that the overall reliability of electronically determined GO-annotations is increasing, but still very low. The mean value of reliability was ≈30% in 2006 and increase to 50% in 2011 (Škunca et al., 2012). The variations are significant among different inference methods, types of annotations, and organisms. Further, functional annotation, which is only based on GO-terms can result in a considerable bias (Schnoes et al., 2013). INDIGO utilizes all InterProScan derived GO-terms whether they are emerging from longer domains such as PFAM, TIGRfam, or PROSITE short consensus patterns. It is common that PROSITE IDs do not relate to any GO term, yet a longer domain in the vicinity or around a PROSITE pattern yields a GO-term associated to a POI. Currently, 11,910 ORFs (10.6%) annotated in INDIGO are associated with a GO-term and a PROSIT ID, which both describe the same function. The INDIGO data warehouse based annotation (AAMG) combines various annotation methods. Unlike other data warehouses, INDIGO keeps and organizes all annotation meta data even if these are not in agreement with the final annotation (Alam et al., 2013). All GO-term and PROSITE IDs, which are available from these meta data are used by the PPM algorithm. In two cases, the PPM algorithm based function predictions differ from the INDIGO annotation. γ-CA identified by the PPM algorithm were previously annotated as “predicted acetyltransferase isoleucine patch superfamily” or “Ferripyochelin binding protein.” Other PPM algorithm based functions narrowed the INDIGO annotation down to only one function. The prephenate DHs were originally annotated as both, Chorismate and Prephenate DH.

In summary both, consensus patterns and GO-terms are standard tools to identify the function of a gene, yet they have weaknesses. The key to increase reliability is combination of descriptors. Since GO-terms (profiles) and PROSITE IDS (patterns) provide orthogonal information of protein function (with the exception of GO-terms based on consensus patterns) selecting combination of both descriptors is a powerful tool to identify the function of a gene product with higher reliability, particularly for novel and distantly related organisms. The PPM algorithm combines those advantages and is able to select for all three combinations of descriptors: the profile sets, the pattern sets and the profile and pattern sets. The strict PPM algorithm extracts and ranks in our case the top 0.1% of most reliably annotated genes. Since genomic data are growing at a much faster pace than experimental verification can proceed, a focus on quality rather than quantity is required. The PPM algorithm guides experimentalists to relevant starting points for successful expression, characterization, and verification of gene products.

### Distantly related sequences from novel organisms

**Figure 4:**
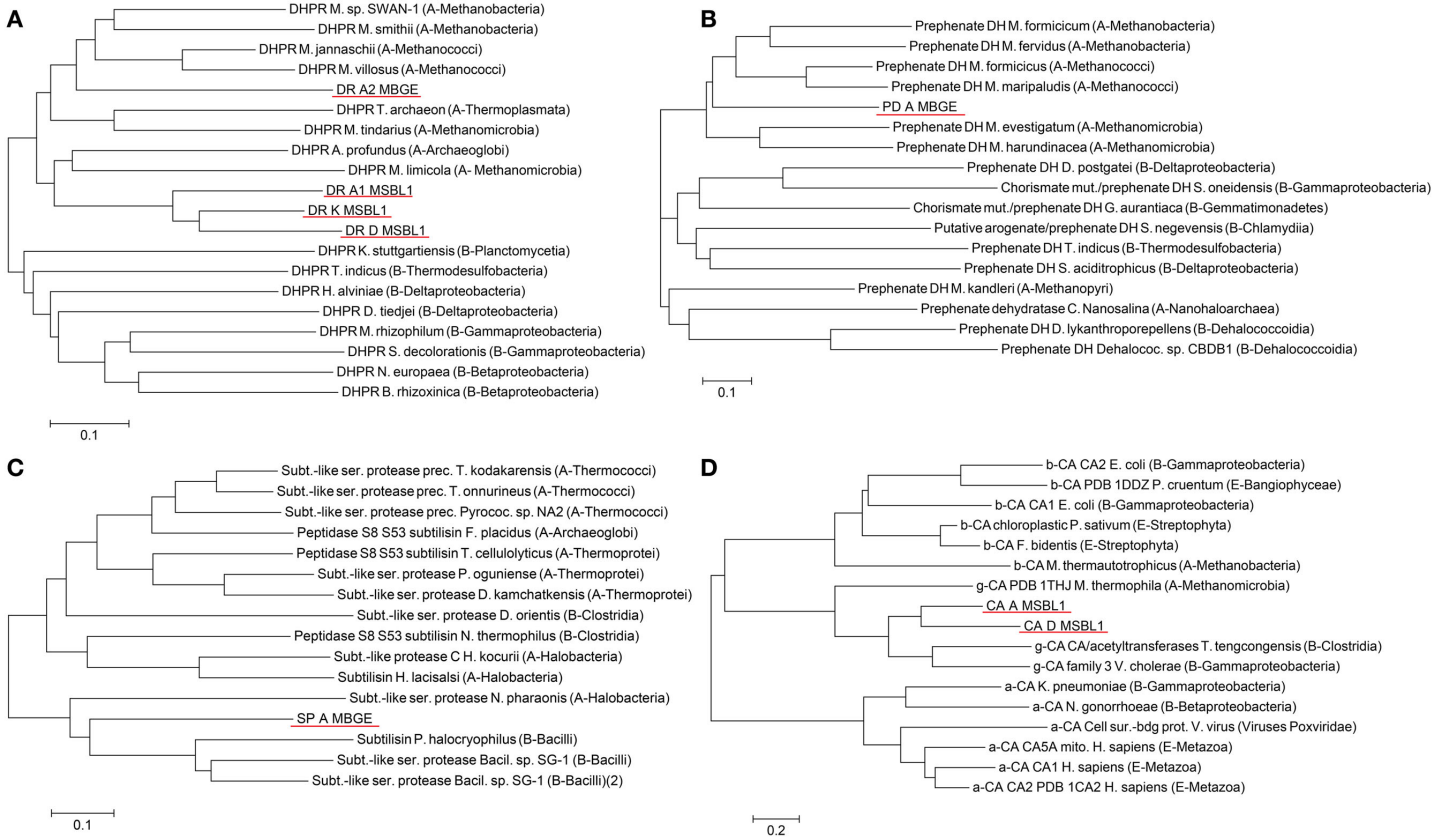
**Analysis of the phylogenetic relationships of the some of the genes, which were identified by PPM analysis as highly reliably, annotated**. PPM profile and pattern hits Dihydrodipicolinate reductases DR_A1, DR_A2, DR_D, and DR_K **(A)**, PPM profile hit prephenate dehydrogenase PD_A **(B)**, PPM pattern hit subtilisin SD_A **(C)** and manual PPM hits γ carbonic anhydrase CA_A and CA_D **(D)**. Scale bars 0.1 (**A–C**) or 0.2 (**D**) amino acid substitutions per site.

Phylogenetic analysis of gene sequences identified as candidates for expression tests revealed a high evolutionary distance to any known sequence (Figure 4). In case of the PPM profile and pattern set hits, which all are DHPRs, the phylogenetic tree with the closest related organisms includes both, the archeal and bacterial domains of life (Figure 4A). The four identified hits are all in the archeal branch. The three hits from the organism MSBL1 (DR_A1, DR_D, and DR_K) are clustering together in a separate branch, connected to *Acheoglobales* and *Methanomicroba*. The hit from the organism MBGE (DR_A2) is in a separate branch and closer related to *Methanobacteria* and *Methanococci*. As indicated by the long branches the junction to the closest previously known sequences occurs at 0.3–0.35 amino acid substitutions per site. The PPM multi-profile hit prephenate dehydrogenase from MBGE (Figure 4B) shows phylogenetic relations similar to DHPR. The closest related enzymes found are from archea and the closest related sequences are from *Methanococci* and *Methanobacteria*. The junction to the closest previously known sequences occurs at 0.33 amino acid substitutions per site. The subtilase type sequence from the PPM multi-pattern hit has a different phylogenetic footprint (Figure 4C). Based on the amino acid sequence the novel subtilisin shows equal evolutionary relations to archea and bacteria, which indicates comparatively low sequence mutations in the two different domains compared to their common ancestor. For the γ-CA hits, which are based on a combination of a new profile and pattern, the phylogenetic tree includes all three known classes of CA (Figure 4D). The tree reveals clearly, that the identified sequences fall into the γ class of CAs with very distant relations to the α and β class. Distant phylogenetic relationships are also found for all other hits, underlining the novelty of the SAGs analyzed (Figures S1–S3).

### Current limitations of the PPM approach

The PPM approach intrinsically leads to a high number of false negatives, because not all protein of interest groups can be translated into GO-terms and PROSITE IDs. During conversion from E.C. numbers to profiles (GO-terms) or pattern (PROSITE ID) about 35 or 81% of the POIs are lost, respectively. This limitation will be overcome through the exponential growth of biological data, which will increase the number and precision of GO-terms and PROSITE IDS. The combination of self-derived profiles and pattern can also enhance/enable PPM analysis, even with comparatively flexible sequences that show individually low reliability, as shown for the γ-CA example. Reducing the rigidity of consensus pattern with a high false negative rate may further help to increase hit rates. However, as discussed above, from an experimentalist point of view false positives are of much higher concern and these can be eliminated very effectively by the PPM approach.

### Outlook and conclusion—the red sea extremophiles as source for novel enzymes with high scientific and industrial potential

For the first time SAGs were used to identify proteins for biotechnological applications. The eleven different genes, which were extracted from the INDIGO database during this study as candidates for expression just give a glimpse of the potential the Red Sea brine pools extremophiles have for discovery of novel enzymes. Not only the great phylogenetic distance to any described organism but also the extreme anoxic, high temperature, and hypersaline environment makes the enzymes of those organisms highly valuable. Enzymatic activity at high temperature and with low water activity can enable biocatalysis to be a tool for complex chemical reactions giving high yield and enantiomeric excess and under conditions that were so far out of reach for biological applications. Investigation of the enzymes, for which genes were identified here, will help understanding the limitations and adaptation of life at such extreme places.

The PPM algorithm is not intended to be a competitor for standard annotation. However, it is a powerful tool to analyze functions of proteins of extremophilic organisms that are only distantly related to organisms described so far. The PPM algorithm helps experimentalists to extract proteins and particularly enzymes with high confidence from databases with only limited annotation reliability, e.g., when SAGs of extremophiles are used.

The combination of orthogonal descriptors may also facilitate screening of other genomic data for proteins of interest, e.g., those resulting from metagenomic or metatranscriptomic sampling as well as from shotgun sequencing. For metagenomic sequences the most reliable functional annotations are achieved using homology-based approaches against publicly available reference sequence databases including GO. Recently, it was recommended for metagenomic data to run a motif-based analysis (e.g., using PROSITE-IDs) in parallel to the homology-based functional prediction (Prakash and Taylor, 2012). The PPM algorithm provides an example using this approach. However, since the PPM algorithm was developed to minimize the number of false positive hits when experimentalists search genomic databases for proteins of interest and we expect also for metagenomic data that the increased reliability of genes identified by this algorithm will be it's main advantage.

The publicly available scripts used in this study (i) AutoTECNo, (ii) PPM processor in combination with (iii) the INDIGO data warehouse are powerful tools, with a minimalistic character to keep handling of extreme large datasets simple. The PPM algorithm will facilitate experimental characterization of extremophilic proteins and therefore help to increase the general understanding of life at extreme conditions and exploiting its biotechnological potential. The enzymes identified in this study will be the first of many proteins on this path.

### Conflict of interest statement

The authors declare that the research was conducted in the absence of any commercial or financial relationships that could be construed as a potential conflict of interest.
