# Supplementary material for: Mining a database of single amplified genomes from Red Sea brine pool extremophiles—improving reliability of gene function prediction using a profile and pattern matching algorithm (PPMA)
Source: Front Microbiol. 2014 Apr 7;5:134. doi: 10.3389/fmicb.2014.00134 (PMC3985023; doi:10.3389/fmicb.2014.00134)
Supplement: Supplementary file 1 [file DataSheet1.DOCX]

Supplementary figures


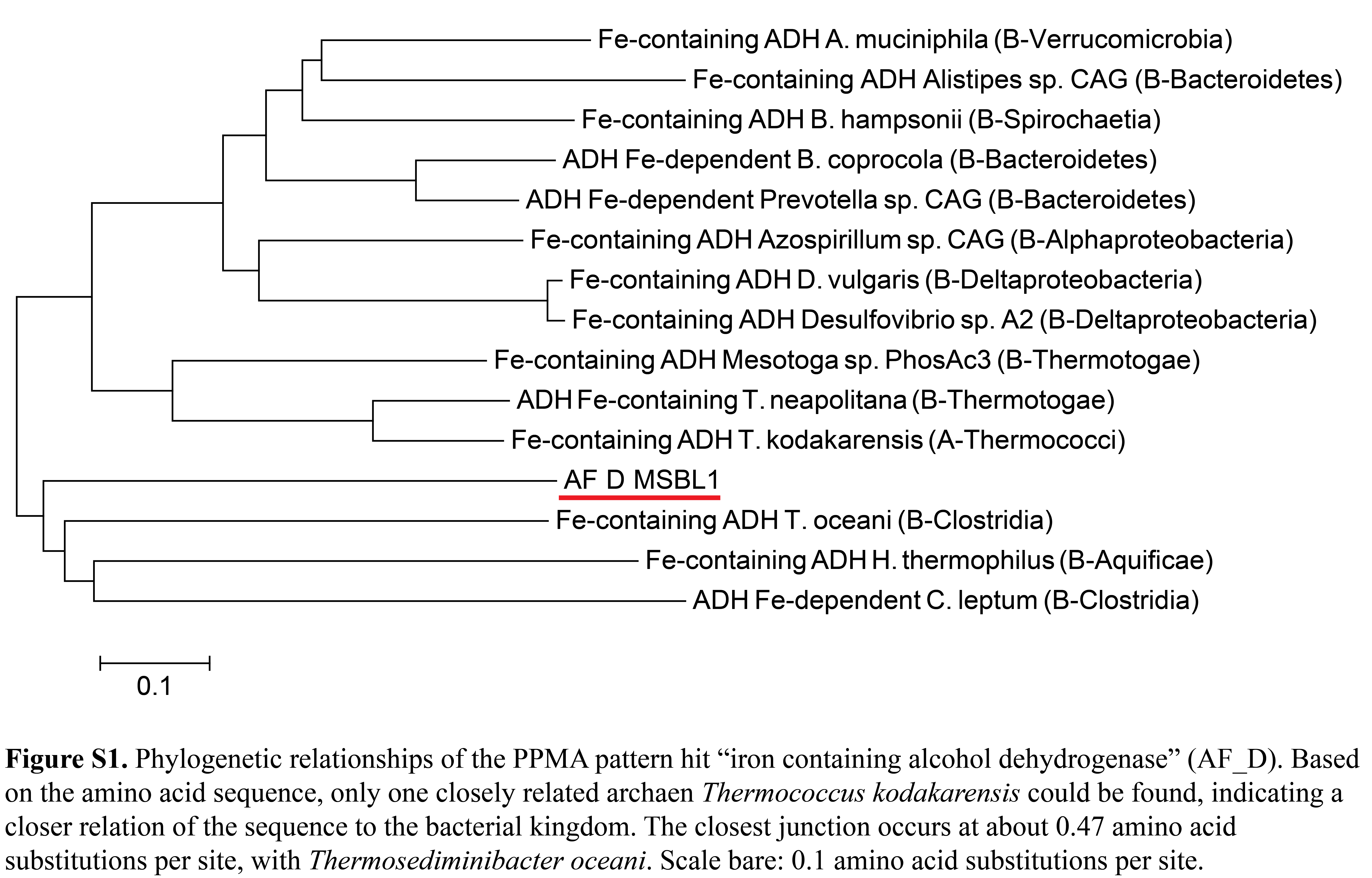


**Figure S1.** Phylogenetic relationships of the PPMA pattern hit “iron containing alcohol dehydrogenase” (AF_D). Based on the amino acid sequence only one closely related archeon *Thermococcus kodakarensis* could be found, indicating a closer relation of the sequence to the bacterial kingdom. The closest junction occurs at about 0.47 amino acid substitutions per site, with *Thermosediminibacter oceani*. Scale bare: 0.1 amino acid substitutions per site.


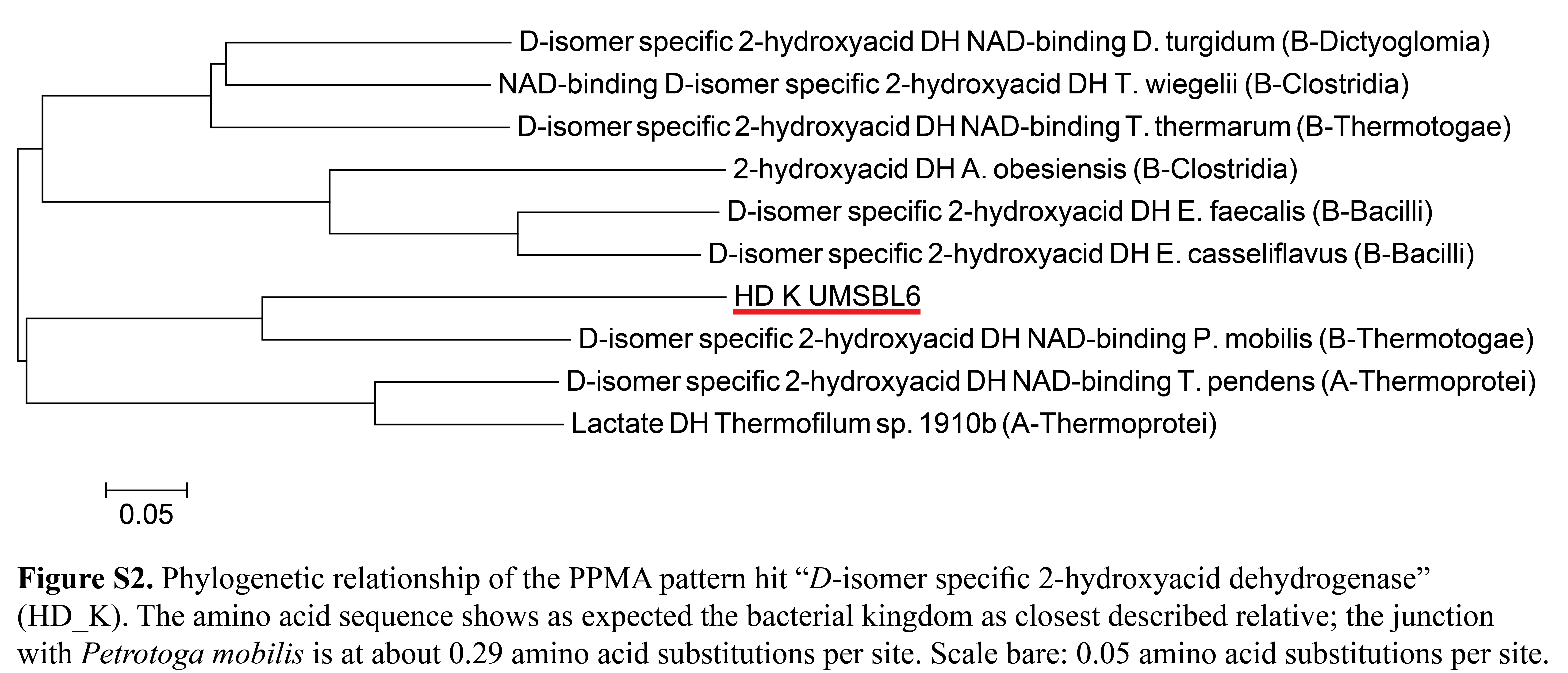


**Figure S2.** Phylogenetic relationships of the PPMA pattern hit “*D*-isomer specific 2-hydroxyacid dehydrogenase” (HD_K). The amino acid sequence shows as expected the bacterial kingdom as closest described relative; the junction with *Petrotoga mobilis* is at about 0.29 amino acid substitutions per site. Scale bare: 0.05 amino acid substitutions per site.

**
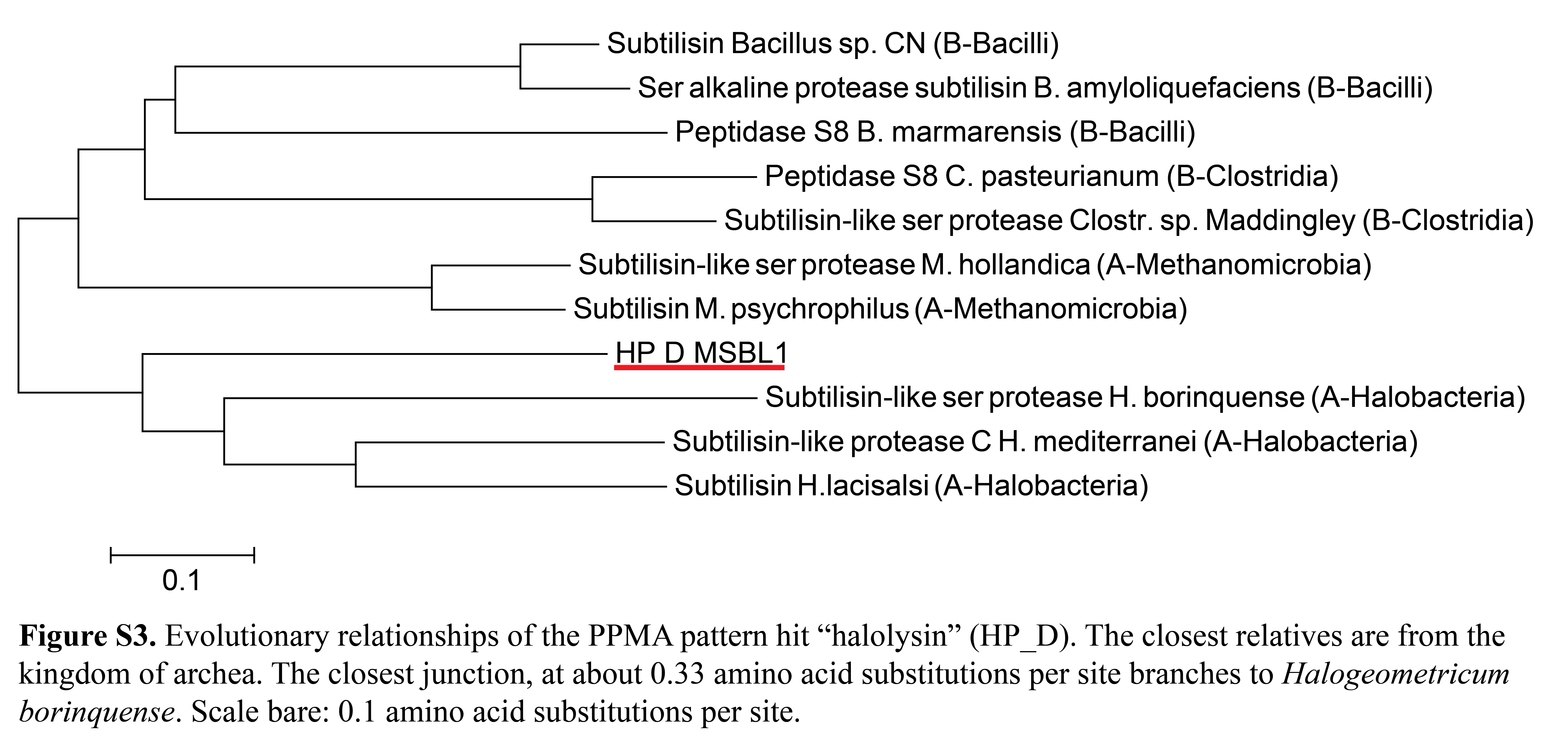
**

**Figure S3.** Evolutionary relationships of the PPMA pattern hit “halolysin” (HP_D). The closest relatives are from the kingdom of archea. The closest junction at about 0.33 amino acid substitutions per site branches to *Halogeometricum borinquense*. Scale bare: 0.1 amino acid substitutions per site
