# Supplementary material for: Mining a database of single amplified genomes from Red Sea brine pool extremophiles—improving reliability of gene function prediction using a profile and pattern matching algorithm (PPMA)
Source: Front Microbiol. 2014 Apr 7;5:134. doi: 10.3389/fmicb.2014.00134 (PMC3985023; doi:10.3389/fmicb.2014.00134)
Supplement: Supplementary file 2 [file DataSheet2.DOCX]

**Table S1. E.C. number coverage of the 15 protein of interest families.**

| **No** | **POI-Group** | **Description** | **E.C. no. range** | **Total E.C. no in class** | **Non- redundant** | **selected** |
| --- | --- | --- | --- | --- | --- | --- |
| 1 | Alcohol DH | Interconversion of alcohols and aldehydes/ketones | 1.1.1.1 - 1.2.99.3 | 101 | 32 | 25 |
| 2 | Formate DH | Conversion of CO_2_ into format | 1.1.5.6 - 1.2.2.3 | 29 | 6 | 6 |
| 3 | Formaldehyde DH | Interconversion of formaldehyde and formate | 1.1.1.1 - 3.1.2.12 | 23 | 9 | 4 |
| 4 | Carbon monoxide DH | Interconversion of CO and CO_2_ | 1.2.2.4 - 6.2.1.1 | 19 | 4 | 4 |
| 5 | Ene reductases | Stereoselective reduction of alkenes | 1.1.1.105 - 1.97.1.8 | 1'162 | 107 | 65 |
| 6 | Protease | Hydrolysis of peptide bonds | 3.1.1.5 - 3.4.99.B1 | 741 | 217 | 111 |
| 7 | Terpene synthase | Synthesis of basic, (mulit-)cyclic terpene structures | 1.14.13.B14 - 5.5.1.8 | 35 | 23 | 17 |
| 8 | Nitrogenase | Fixation of nitrogen from air | 1.18.6.1 - 3.2.2.24 | 18 | 4 | 2 |
| 9 | Lipase | Hydrolysis of triglyceride esters | 3.1.1.1 - 4.6.1.14 | 380 | 26 | 25 |
| 10 | Carbonic anhydrase | Interconversion of CO_2_ and Bicarbonate | 4.2.1.1 | 58 | 1 | 1 |
| 11 | Acetylene hydratase | Synthesis of aldehydes from acetylene | 4.2.1.112 | 2 | 1 | 1 |
| 12 | Acetyl-CoA synthetase | Activation of acetate for further conversion | 6.1.1.X | 8 | 3 | 3 |
| 13 | pylRS | Aminoacyl tRNA synthetase, acting on pyrrolysin | 6.1.1.26 | 1 | 1 | 1 |
| 14 | pyltRNA | tRNA coding for pyrrolysine (22^nd^ amino acid) | - | 0 | 0 | 0 |
| 15 | Aquaporins | Integral membrane proteins controlling osmotic pressure | - | 0 | 0 | 0 |
| **Total** | | | | **2'577** | **433** | **264** |
