# Supplementary material for: Mining a database of single amplified genomes from Red Sea brine pool extremophiles—improving reliability of gene function prediction using a profile and pattern matching algorithm (PPMA)
Source: Front Microbiol. 2014 Apr 7;5:134. doi: 10.3389/fmicb.2014.00134 (PMC3985023; doi:10.3389/fmicb.2014.00134)
Supplement: Supplementary file 3 [file DataSheet3.DOCX]

**Table S2. All 265 E.C. numbers selected as input for the PPM algorithm**

| **Type** | **E.C. No** | **Recommended Name** |
| --- | --- | --- |
| ADH | 1.1.1.1 | alcohol dehydrogenase |
|  | 1.1.1.2 | alcohol dehydrogenase (NADP+) |
|  | 1.1.1.54 | allyl-alcohol dehydrogenase |
|  | 1.1.1.71 | alcohol dehydrogenase [NAD(P)+] |
|  | 1.1.1.90 | aryl-alcohol dehydrogenase |
|  | 1.1.1.91 | aryl-alcohol dehydrogenase (NADP+) |
|  | 1.1.1.97 | 3-hydroxybenzyl-alcohol dehydrogenase |
|  | 1.1.1.144 | perillyl-alcohol dehydrogenase |
|  | 1.1.1.192 | long-chain-alcohol dehydrogenase |
|  | 1.1.1.194 | coniferyl-alcohol dehydrogenase |
|  | 1.1.1.195 | cinnamyl-alcohol dehydrogenase |
|  | 1.1.1.245 | cyclohexanol dehydrogenase |
|  | 1.1.1.257 | 4-(hydroxymethyl)benzenesulfonate dehydrogenase |
|  | 1.1.1.265 | 3-methylbutanal reductase |
|  | 1.1.1.284 | S-(hydroxymethyl)glutathione dehydrogenase |
|  | 1.1.1.314 | germacrene A alcohol dehydrogenase |
|  | 1.1.2.6 | polyvinyl alcohol dehydrogenase (cytochrome) |
|  | 1.1.2.7 | methanol dehydrogenase (cytochrome c) |
|  | 1.1.2.8 | alcohol dehydrogenase (cytochrome c) |
|  | 1.1.5.5 | alcohol dehydrogenase (quinone) |
|  | 1.1.5.7 | cyclic alcohol dehydrogenase (quinone) |
|  | 1.1.9.1 | alcohol dehydrogenase (azurin) |
|  | 1.1.99.24 | hydroxyacid-oxoacid transhydrogenase |
|  | 1.1.99.36 | NDMA-dependent alcohol dehydrogenase |
|  | 1.2.99.3 | aldehyde dehydrogenase (pyrroloquinoline-quinone) |
| Formate_DH | 1.1.5.6 | formate dehydrogenase-N |
|  | 1.1.99.33 | formate dehydrogenase (acceptor) |
|  | 1.2.1.2 | formate dehydrogenase |
|  | 1.2.1.43 | formate dehydrogenase (NADP+) |
|  | 1.2.2.1 | formate dehydrogenase (cytochrome) |
|  | 1.2.2.3 | formate dehydrogenase (cytochrome-c-553) |
| Formaldehyde_DH | 1.1.1.284 | S-(hydroxymethyl)glutathione dehydrogenase |
|  | 1.1.1.306 | S-(hydroxymethyl)mycothiol dehydrogenase |
|  | 1.2.1.46 | formaldehyde dehydrogenase |
|  | 3.1.2.12 | S-formylglutathione hydrolase |
| CO-DH | 1.2.7.4 | carbon-monoxide dehydrogenase (ferredoxin) |
|  | 1.2.99.2 | carbon-monoxide dehydrogenase (acceptor) |
|  | 2.3.1.169 | CO-methylating acetyl-CoA synthase |
|  | 6.2.1.1 | acetate-CoA ligase |
| Ene-Reductase | 1.1.1.252 | tetrahydroxynaphthalene reductase |
|  | 1.3.1.1 | dihydropyrimidine dehydrogenase (NAD+) |
|  | 1.3.1.10 | enoyl-[acyl-carrier-protein] reductase (NADPH, B-specific) |
|  | 1.3.1.11 | 2-coumarate reductase |
|  | 1.3.1.12 | prephenate dehydrogenase |
|  | 1.3.1.13 | prephenate dehydrogenase (NADP+) |
|  | 1.3.1.16 | beta-nitroacrylate reductase |
|  | 1.3.1.17 | 3-methyleneoxindole reductase |
|  | 1.3.1.19 | cis-1,2-dihydrobenzene-1,2-diol dehydrogenase |
|  | 1.3.1.2 | dihydropyrimidine dehydrogenase (NADP+) |
|  | 1.3.1.20 | trans-1,2-dihydrobenzene-1,2-diol dehydrogenase |
|  | 1.3.1.25 | 1,6-dihydroxycyclohexa-2,4-diene-1-carboxylate dehydrogenase |
|  | 1.3.1.26 | dihydrodipicolinate reductase |
|  | 1.3.1.27 | 2-hexadecenal reductase |
|  | 1.3.1.28 | 2,3-dihydro-2,3-dihydroxybenzoate dehydrogenase |
|  | 1.3.1.29 | cis-1,2-dihydro-1,2-dihydroxynaphthalene dehydrogenase |
|  | 1.3.1.31 | 2-enoate reductase |
|  | 1.3.1.32 | maleylacetate reductase |
|  | 1.3.1.34 | 2,4-dienoyl-CoA reductase (NADPH) |
|  | 1.3.1.37 | cis-2-enoyl-CoA reductase (NADPH) |
|  | 1.3.1.38 | trans-2-enoyl-CoA reductase (NADPH) |
|  | 1.3.1.39 | enoyl-[acyl-carrier-protein] reductase (NADPH, A-specific) |
|  | 1.3.1.40 | 2-hydroxy-6-oxo-6-phenylhexa-2,4-dienoate reductase |
|  | 1.3.1.42 | 12-oxophytodienoate reductase |
|  | 1.3.1.43 | arogenate dehydrogenase |
|  | 1.3.1.44 | trans-2-enoyl-CoA reductase (NAD+) |
|  | 1.3.1.45 | 2'-hydroxyisoflavone reductase |
|  | 1.3.1.49 | cis-3,4-dihydrophenanthrene-3,4-diol dehydrogenase |
|  | 1.3.1.52 | 2-methyl-branched-chain-enoyl-CoA reductase |
|  | 1.3.1.53 | (3S,4R)-3,4-dihydroxycyclohexa-1,5-diene-1,4-dicarboxylate dehydrogenase |
|  | 1.3.1.56 | cis-2,3-dihydrobiphenyl-2,3-diol dehydrogenase |
|  | 1.3.1.58 | 2,3-dihydroxy-2,3-dihydro-p-cumate dehydrogenase |
|  | 1.3.1.6 | fumarate reductase (NADH) |
|  | 1.3.1.60 | dibenzothiophene dihydrodiol dehydrogenase |
|  | 1.3.1.63 | 2,4-dichlorobenzoyl-CoA reductase |
|  | 1.3.1.64 | phthalate 4,5-cis-dihydrodiol dehydrogenase |
|  | 1.3.1.65 | 5,6-dihydroxy-3-methyl-2-oxo-1,2,5,6-tetrahydroquinoline dehydrogenase |
|  | 1.3.1.66 | cis-dihydroethylcatechol dehydrogenase |
|  | 1.3.1.67 | cis-1,2-dihydroxy-4-methylcyclohexa-3,5-diene-1-carboxylate dehydrogenase |
|  | 1.3.1.68 | 1,2-dihydroxy-6-methylcyclohexa-3,5-dienecarboxylate dehydrogenase |
|  | 1.3.1.69 | zeatin reductase |
|  | 1.3.1.7 | meso-tartrate dehydrogenase |
|  | 1.3.1.73 | 1,2-dihydrovomilenine reductase |
|  | 1.3.1.74 | 2-alkenal reductase |
|  | 1.3.1.75 | divinyl chlorophyllide a 8-vinyl-reductase |
|  | 1.3.1.76 | precorrin-2 dehydrogenase |
|  | 1.3.1.77 | anthocyanidin reductase |
|  | 1.3.1.78 | arogenate dehydrogenase (NADP+) |
|  | 1.3.1.79 | arogenate dehydrogenase [NAD(P)+] |
|  | 1.3.1.8 | acyl-CoA dehydrogenase (NADP+) |
|  | 1.3.1.81 | (+)-pulegone reductase |
|  | 1.3.1.82 | (-)-isopiperitenone reductase |
|  | 1.3.1.83 | geranylgeranyl diphosphate reductase |
|  | 1.3.1.84 | acrylyl-CoA reductase (NADPH) |
|  | 1.3.1.85 | crotonyl-CoA carboxylase/reductase |
|  | 1.3.1.86 | crotonyl-CoA reductase |
|  | 1.3.1.87 | 3-(cis-5,6-dihydroxycyclohexa-1,3-dien-1-yl)propanoate dehydrogenase |
|  | 1.3.1.9 | enoyl-[acyl-carrier-protein] reductase (NADH) |
|  | 1.3.8.1 | butyryl-CoA dehydrogenase |
|  | 1.6.5.5 | NADPH:quinone reductase |
|  | 1.6.99.1 | NADPH dehydrogenase |
|  | 1.7.1.6 | azobenzene reductase |
|  | 1.7.1.11 | 4-(dimethylamino)phenylazoxybenzene reductase |
|  | 1.7.1.12 | N-hydroxy-2-acetamidofluorene reductase |
|  | 1.97.1.8 | tetrachloroethene reductive dehalogenase |
| Protease | 3.4.11.6 | aminopeptidase B |
|  | 3.4.14.9 | tripeptidyl-peptidase I |
|  | 3.4.16.2 | lysosomal Pro-Xaa carboxypeptidase |
|  | 3.4.16.6 | carboxypeptidase D |
|  | 3.4.19.12 | ubiquitinyl hydrolase 1 |
|  | 3.4.21.1 | chymotrypsin |
|  | 3.4.21.3 | metridin |
|  | 3.4.21.4 | trypsin |
|  | 3.4.21.10 | acrosin |
|  | 3.4.21.12 | alpha-lytic endopeptidase |
|  | 3.4.21.19 | glutamyl endopeptidase |
|  | 3.4.21.21 | coagulation factor VIIa |
|  | 3.4.21.25 | cucumisin |
|  | 3.4.21.26 | prolyl oligopeptidase |
|  | 3.4.21.27 | coagulation factor XIa |
|  | 3.4.21.32 | brachyurin |
|  | 3.4.21.35 | tissue kallikrein |
|  | 3.4.21.37 | leukocyte elastase |
|  | 3.4.21.39 | chymase |
|  | 3.4.21.41 | complement subcomponent C1r |
|  | 3.4.21.42 | complement subcomponent C1s |
|  | 3.4.21.46 | complement factor D |
|  | 3.4.21.50 | lysyl endopeptidase |
|  | 3.4.21.53 | Endopeptidase La |
|  | 3.4.21.59 | Tryptase |
|  | 3.4.21.61 | Kexin |
|  | 3.4.21.62 | Subtilisin |
|  | 3.4.21.63 | Oryzin |
|  | 3.4.21.70 | Pancreatic endopeptidase E |
|  | 3.4.21.72 | IgA-specific serine endopeptidase |
|  | 3.4.21.74 | Venombin A |
|  | 3.4.21.75 | Furin |
|  | 3.4.21.76 | Myeloblastin |
|  | 3.4.21.78 | Granzyme A |
|  | 3.4.21.79 | Granzyme B |
|  | 3.4.21.80 | Streptogrisin A |
|  | 3.4.21.81 | Streptogrisin B |
|  | 3.4.21.82 | Glutamyl endopeptidase II |
|  | 3.4.21.83 | Oligopeptidase B |
|  | 3.4.21.89 | Signal peptidase I |
|  | 3.4.21.90 | Togavirin |
|  | 3.4.21.91 | Flavivirin |
|  | 3.4.21.92 | Endopeptidase Clp |
|  | 3.4.21.93 | Proprotein convertase 1 |
|  | 3.4.21.97 | assemblin |
|  | 3.4.21.98 | hepacivirin |
|  | 3.4.21.102 | C-terminal processing peptidase |
|  | 3.4.21.104 | mannan-binding lectin-associated serine protease-2 |
|  | 3.4.21.107 | peptidase Do |
|  | 3.4.21.108 | HtrA2 peptidase |
|  | 3.4.21.112 | site-1 protease |
|  | 3.4.21.120 | oviductin |
|  | 3.4.22.7 | asclepain |
|  | 3.4.22.8 | clostripain |
|  | 3.4.22.10 | streptopain |
|  | 3.4.22.14 | actinidain |
|  | 3.4.22.28 | picornain 3C |
|  | 3.4.22.29 | picornain 2A |
|  | 3.4.22.34 | Legumain |
|  | 3.4.22.35 | Histolysain |
|  | 3.4.22.37 | gingipain R |
|  | 3.4.22.39 | adenain |
|  | 3.4.22.40 | bleomycin hydrolase |
|  | 3.4.22.44 | nuclear-inclusion-a endopeptidase |
|  | 3.4.22.45 | helper-component proteinase |
|  | 3.4.22.46 | L-peptidase |
|  | 3.4.22.47 | gingipain K |
|  | 3.4.22.48 | staphopain |
|  | 3.4.22.50 | V-cath endopeptidase |
|  | 3.4.22.51 | cruzipain |
|  | 3.4.22.66 | calicivirin |
|  | 3.4.22.67 | zingipain |
|  | 3.4.22.68 | Ulp1 peptidase |
|  | 3.4.22.69 | SARS coronavirus main proteinase |
|  | 3.4.23.12 | nepenthesin |
|  | 3.4.23.18 | Aspergillopepsin I |
|  | 3.4.23.20 | Penicillopepsin |
|  | 3.4.23.21 | Rhizopuspepsin |
|  | 3.4.23.22 | Endothiapepsin |
|  | 3.4.23.23 | Mucorpepsin |
|  | 3.4.23.24 | Candidapepsin |
|  | 3.4.23.25 | Saccharopepsin |
|  | 3.4.23.26 | Rhodotorulapepsin |
|  | 3.4.23.29 | Polyporopepsin |
|  | 3.4.23.39 | plasmepsin II |
|  | 3.4.23.40 | Phytepsin |
|  | 3.4.23.41 | yapsin 1 |
|  | 3.4.23.45 | memapsin 1 |
|  | 3.4.23.46 | memapsin 2 |
|  | 3.4.23.48 | plasminogen activator Pla |
|  | 3.4.23.49 | omptin |
|  | 3.4.24.18 | meprin A |
|  | 3.4.24.23 | matrilysin |
|  | 3.4.24.25 | vibriolysin |
|  | 3.4.24.26 | pseudolysin |
|  | 3.4.24.27 | thermolysin |
|  | 3.4.24.28 | bacillolysin |
|  | 3.4.24.29 | aureolysin |
|  | 3.4.24.32 | beta-Lytic metalloendopeptidase |
|  | 3.4.24.36 | leishmanolysin |
|  | 3.4.24.37 | saccharolysin |
|  | 3.4.24.38 | gametolysin |
|  | 3.4.24.40 | serralysin |
|  | 3.4.24.50 | bothrolysin |
|  | 3.4.24.55 | pitrilysin |
|  | 3.4.24.63 | meprin B |
|  | 3.4.24.69 | bontoxilysin |
|  | 3.4.24.77 | snapalysin |
|  | 3.4.24.78 | gpr endopeptidase |
|  | 3.4.24.85 | S2P endopeptidase |
|  | 3.4.25.2 | HslU-HslV peptidase |
| Terpene_Synthase | 4.2.3.22 | germacradienol synthase |
|  | 4.2.3.23 | germacrene-A synthase |
|  | 4.2.3.46 | alpha-farnesene synthase |
|  | 4.2.3.47 | beta-farnesene synthase |
|  | 4.2.3.49 | (3R,6E)-nerolidol synthase |
|  | 4.2.3.51 | beta-phellandrene synthase (neryl-diphosphate-cyclizing) |
|  | 4.2.3.6 | trichodiene synthase |
|  | 4.2.3.7 | pentalenene synthase |
|  | 4.2.3.9 | aristolochene synthase |
|  | 4.2.3.14 | pinene synthase |
|  | 4.2.3.36 | terpentetriene synthase |
|  | 4.2.3.39 | epi-cedrol synthase |
|  | 4.2.3.73 | valencene synthase |
|  | 4.2.3.90 | 5-epi-alpha-selinene synthase |
|  | 5.4.99.34 | germanicol synthase |
|  | 5.4.99.35 | taraxerol synthase |
|  | 5.5.1.8 | bornyl diphosphate synthase |
| Nitrogenase | 1.18.6.1 | nitrogenase |
|  | 1.19.6.1 | nitrogenase (flavodoxin) |
| Lipase | 3.1.1.1 | carboxylesterase |
|  | 3.1.1.2 | arylesterase |
|  | 3.1.1.3 | triacylglycerol lipase |
|  | 3.1.1.4 | phospholipase A2 |
|  | 3.1.1.5 | lysophospholipase |
|  | 3.1.1.13 | sterol esterase |
|  | 3.1.1.23 | acylglycerol lipase |
|  | 3.1.1.26 | galactolipase |
|  | 3.1.1.32 | phospholipase A1 |
|  | 3.1.1.34 | lipoprotein lipase |
|  | 3.1.1.47 | 1-alkyl-2-acetylglycerophosphocholine esterase |
|  | 3.1.1.52 | phosphatidylinositol deacylase |
|  | 3.1.1.79 | hormone-sensitive lipase |
|  | 3.1.2.2 | palmitoyl-CoA hydrolase |
|  | 3.1.2.22 | palmitoyl[protein] hydrolase |
|  | 3.1.4.3 | phospholipase C |
|  | 3.1.4.4 | phospholipase D |
|  | 3.1.4.11 | phosphoinositide phospholipase C |
|  | 3.1.4.12 | sphingomyelin phosphodiesterase |
|  | 3.1.4.39 | alkylglycerophosphoethanolamine phosphodiesterase |
|  | 3.1.4.41 | sphingomyelin phosphodiesterase D |
|  | 3.1.4.50 | glycosylphosphatidylinositol phospholipase D |
|  | 3.1.4.54 | N-acetylphosphatidylethanolamine-hydrolysing phospholipase D |
|  | 4.6.1.13 | phosphatidylinositol diacylglycerol-lyase |
|  | 4.6.1.14 | glycosylphosphatidylinositol diacylglycerol-lyase |
| Carbonic Anhydrase | 4.2.1.1 | carbonate dehydratase |
| Acetylene hydratase | 4.2.1.112 | acetylene hydratase |
| AcetylCoA_Synthetase | 6.2.1.1 | acetate-CoA ligase |
|  | 6.2.1.13 | Acetate-CoA ligase (ADP-forming) |
|  | 6.2.1.16 | Acetoacetate-CoA ligase |
| PylRS | 6.1.1.26 | pyrrolysine-tRNAPyl ligase |
