# Supplementary material for: Mining a database of single amplified genomes from Red Sea brine pool extremophiles—improving reliability of gene function prediction using a profile and pattern matching algorithm (PPMA)
Source: Front Microbiol. 2014 Apr 7;5:134. doi: 10.3389/fmicb.2014.00134 (PMC3985023; doi:10.3389/fmicb.2014.00134)
Supplement: Supplementary file 4 [file DataSheet4.DOCX]

**Table S3. The 171 GO terms used for the profile analysis**

| **GO-Term** | **Type** | **Definition** | **Source** |
| --- | --- | --- | --- |
| GO:0004025 | ADH | Catalysis of the reaction: an alcohol + NAD+ = an aldehyde or ketone + NADH + H+, requiring the presence of iron. | EC:1.1.1.1 |
| GO:0008106 |  | Catalysis of the reaction: an alcohol + NADP+ = an aldehyde + NADPH + H+. | EC:1.1.1.2 |
| GO:0047655 |  | Catalysis of the reaction: allyl alcohol + NADP(+) = acrolein + H(+) + NADPH | EC:1.1.1.54 |
| GO:0018455 |  | Catalysis of the reaction: an alcohol + NAD(P)+ = an aldehyde + NAD(P)H + H+. | EC:1.1.1.71 |
| GO:0018456 |  | Catalysis of the reaction: an aromatic alcohol + NAD+ = an aromatic aldehyde + NADH + H+. | EC:1.1.1.90 |
| GO:0047681 |  | Catalysis of the reaction: an aromatic alcohol + NADP+ = an aromatic aldehyde + NADPH. | EC:1.1.1.91 |
| GO:0047048 |  | Catalysis of the reaction: 3-hydroxybenzyl alcohol + NADP(+) = 3-hydroxybenzaldehyde + H(+) + NADPH. | EC:1.1.1.97 |
| GO:0018457 |  | Catalysis of the reaction: NAD(+) + perillyl alcohol = H(+) + NADH + perillyl aldehyde. | EC:1.1.1.144 |
| GO:0050060 |  | Catalysis of the reaction: a long-chain alcohol + 2 NAD+ + H2O = a long-chain carboxylate + 2 NADH. | EC:1.1.1.192 |
| GO:0050268 |  | Catalysis of the reaction: coniferyl alcohol + NADP+ = coniferyl aldehyde + NADPH. | EC:1.1.1.194 |
| GO:0052747 |  | Catalysis of the reaction: sinapaldehyde + NADPH + H+ = sinapyl-alcohol + NADP+. | EC:1.1.1.195 |
| GO:0018460 |  | Catalysis of the reaction: cyclohexanol + NAD+ = cyclohexanone + NADH + H+. | EC:1.1.1.245 |
| GO:0018462 |  | Catalysis of the reaction: 4-(hydroxymethyl)benzenesulfonate + NAD(+) = 4-formylbenzenesulfonate + H(+) + NADH. | EC:1.1.1.257 |
| GO:0052676 |  | Catalysis of the reaction: 3-methylbutanol + NAD+ = 3-methylbutanal + NADH + H+. 3-methylbutanal is also known as isovaleraldehyde. | EC:1.1.1.265 |
| GO:0051903 |  | Catalysis of the reaction: S-(hydroxymethyl)glutathione + NAD(P)+ = S-formylglutathione + NAD(P)H + H+. | EC:1.1.1.284 |
| GO:0047059 |  | Catalysis of the reaction: polyvinyl alcohol + ferricytochrome c = oxidized polyvinyl alcohol + ferrocytochrome c + H+. | EC:1.1.2.6 |
| GO:0052933 |  | Catalysis of the reaction: primary alcohol + 2 cytochrome c(L) = 2 reduced cytochrome c(L) + an aldehyde + 2 H+. | EC:1.1.2.7 |
| GO:0052936 |  | Catalysis of the reaction: 2-chloroethanol + 2 cytochrome c = chloroacetaldehyde + 2 reduced cytochrome c. | EC:1.1.2.8 |
| GO:0047988 |  | Catalysis of the reaction: (S)-3-hydroxybutanoate + 2-oxoglutarate = acetoacetate + (R)-2-hydroxyglutarate. | EC:1.1.99.24 |
| GO:0033717 |  | Catalysis of the reaction: D-gluconate + acceptor = 2-dehydro-D-gluconate + reduced acceptor. | EC:1.1.99.3 |
| GO:0009326 | Formate_DH | An enzyme complex that catalyzes the dehydrogenation of formate to produce carbon dioxide (CO2). | EC:1.2.1.2 |
| GO:0047899 |  | Catalysis of the reaction: formate + NADP(+) = CO(2) + NADPH. | EC:1.2.1.43 |
| GO:0047898 |  | Catalysis of the reaction: formate + ferricytochrome b1 = CO2 + ferrocytochrome b1. | EC:1.2.2.1 |
| GO:0047111 |  | Catalysis of the reaction: ferricytochrome C-553 + formate = ferrocytochrome C-553 + CO2. | EC:1.2.2.3 |
| GO:0018467 | Formaldehyde_DH | Catalysis of the reaction: formaldehyde + H(2)O + NAD(+) = formate + 2 H(+) + NADH. | EC:1.2.1.46 |
| GO:0018738 |  | Catalysis of the reaction: S-formylglutathione + H(2)O = formate + glutathione + H(+). | EC:3.1.2.12 |
| GO:0043885 | CO-DH | Catalysis of the reaction: CO + H2O + oxidized ferredoxin = CO2 + reduced ferredoxin. | EC:1.2.7.4 |
| GO:0018492 |  | Catalysis of the reaction: CO + H2O + acceptor = CO2 + reduced acceptor. | EC:1.2.99.2 |
| GO:0043884 |  | Catalysis of the reaction: acetyl-CoA + corrinoid protein = CO + methylcorrinoid protein + CoA. | EC:2.3.1.16 |
| GO:0003987 |  | Catalysis of the reaction: ATP + acetate + CoA = AMP + diphosphate + acetyl-CoA. | EC:6.2.1.1 |
| GO:0047039 | Ene-Reductase | Catalysis of the reaction: NADP+ + scytalone = NADPH + H+ + 1,3,6,8-naphthalenetetrol. | EC:1.1.1.252 |
| GO:0004159 |  | Catalysis of the reaction: 5,6-dihydrouracil + NAD+ = uracil + NADH + H+. | EC:1.3.1.1 |
| GO:0004319 |  | Catalysis of the reaction: acyl-[acyl-carrier protein] + NADP+ = trans-2,3-dehydroacyl-[acyl-carrier protein] + NADPH + H+. | EC:1.3.1.10 |
| GO:0047788 |  | Catalysis of the reaction: 3-(2-hydroxyphenyl)propanoate + NAD(+) = trans-2-coumarate + H(+) + NADH. | EC:1.3.1.11 |
| GO:0008977 |  | Catalysis of the reaction: NAD(+) + prephenate = (4-hydroxyphenyl)pyruvate + CO(2) + NADH. | EC:1.3.1.12 |
| GO:0004665 |  | Catalysis of the reaction: NADP(+) + prephenate = (4-hydroxyphenyl)pyruvate + CO(2) + NADPH. | EC:1.3.1.13 |
| GO:0047703 |  | Catalysis of the reaction: 3-nitropropanoate + NADP(+) = 3-nitroacrylate + H(+) + NADPH. | EC:1.3.1.16 |
| GO:0047567 |  | Catalysis of the reaction: 3-nitropropanoate + NADP(+) = 3-nitroacrylate + H(+) + NADPH. | EC:1.3.1.16 |
| GO:0018504 |  | Catalysis of the reaction: cis-1,2-dihydrobenzene-1,2-diol + NAD+ = catechol + NADH + H+. | EC:1.3.1.19 |
| GO:0017113 |  | Catalysis of the reaction: 5,6-dihydrouracil + NADP+ = uracil + NADPH + H+. | EC:1.3.1.2 |
| GO:0047115 |  | Catalysis of the reaction: NADP+ + trans-1,2-dihydrobenzene-1,2-diol = NADPH + catechol. | EC:1.3.1.20 |
| GO:0047116 |  | Catalysis of the reaction: NAD+ + 1,6-dihydroxycyclohexa-2,4-diene-1-carboxylate = NADH + CO2 + catechol. | EC:1.3.1.25 |
| GO:0008839 |  | Catalysis of the reaction: (S)-2,3,4,5-tetrahydropyridine-2,6-dicarboxylate + NAD(P)+ + H2O = (2S,4S)-4-hydroxy-2,3,4,5-tetrahydrodipicolinate + NAD(P)H + H+. | EC:1.17.1.8 |
| GO:0047543 |  | Catalysis of the reaction: NADP(+) + palmitaldehyde = trans-hexadec-2-enal + H(+) + NADPH. | EC:1.3.1.27 |
| GO:0008667 |  | Catalysis of the reaction: (2S,3S)-2,3-dihydroxy-2,3-dihydrobenzoate + NAD(+) = 2,3-dihydroxybenzoate + H(+) + NADH. | EC:1.3.1.28 |
| GO:0018505 |  | Catalysis of the reaction: cis-1,2-dihydronaphthalene-1,2-diol + NAD+ = naphthalene-1,2-diol + NADH + H+. | EC:1.3.1.29 |
| GO:0047540 |  | Catalysis of the reaction: butanoate + NAD+ = 2-butenoate + NADH + H+. | EC:1.3.1.31 |
| GO:0018506 |  | Catalysis of the reaction: 3-oxoadipate + NAD(P)+ = 2-maleylacetate + NAD(P)H + H+. | EC:1.3.1.32 |
| GO:0008670 |  | Catalysis of the reaction: trans-2,3-didehydroacyl-CoA + NADP+ = trans,trans-2,3,4,5-tetradehydroacyl-CoA + NADPH + H+. | EC:1.3.1.34 |
| GO:0047774 |  | Catalysis of the reaction: acyl-CoA + NADP+ = cis-2,3-dehydroacyl-CoA + NADPH. | EC:1.3.1.37 |
| GO:0019166 |  | Catalysis of the reaction: acyl-CoA + NADP+ = trans-2,3-dehydroacyl-CoA + NADPH + H+. | EC:1.3.1.38 |
| GO:0047117 |  | Catalysis of the reaction: acyl-[acyl-carrier protein] + NADP+ = trans-D2-enoyl-[acyl-carrier protein] + NADPH + H+. | EC:1.3.1.39 |
| GO:0047118 |  | Catalysis of the reaction: 2,6-dioxo-6-phenylhexanoate + NADP(+) = 2-hydroxy-6-oxo-6-phenylhexa-2,4-dienoate + H(+) + NADPH. | EC:1.3.1.40 |
| GO:0016629 |  | Catalysis of the reaction: 8-[(1R,2R)-3-oxo-2-{(Z)-pent-2-en-1-yl}cyclopentyl]octanoate + NADP(+) = (15Z)-12-oxophyto-10,15-dienoate + H(+) + NADPH. | EC:1.3.1.42 |
| GO:0047794 |  | Catalysis of the reaction: L-arogenate + NAD+ = L-tyrosine + NADH + CO2. | EC:1.3.1.43 |
| GO:0050343 |  | Catalysis of the reaction: acyl-CoA + NAD+ = trans-didehydroacyl-CoA + NADH. | EC:1.3.1.44 |
| GO:0047526 |  | Catalysis of the reaction: vestitone + NADP+ = 2'-hydroxyformononetin + NADPH + H+. | EC:1.3.1.45 |
| GO:0018507 |  | Catalysis of the reaction: (3S,4R)-3,4-dihydrophenanthrene-3,4-diol + NAD(+) = H(+) + NADH + phenanthrene-3,4-diol. | EC:1.3.1.49 |
| GO:0047119 |  | Catalysis of the reaction: 2-methylbutanoyl-CoA + NAD(+) = 2-methylbut-2-enoyl-CoA + H(+) + NADH. | EC:1.3.1.52 |
| GO:0047120 |  | Catalysis of the reaction: (3S,4R)-3,4-dihydroxycyclohexa-1,5-diene-1,4-dicarboxylate + NAD(+) = 3,4-dihydroxybenzoate + CO(2) + NADH. | EC:1.3.1.53 |
| GO:0018509 |  | Catalysis of the reaction: cis-3-phenylcyclohexa-3,5-diene-1,2-diol + NAD+ = biphenyl-2,3-diol + NADH + H+. | EC:1.3.1.56 |
| GO:0018511 |  | Catalysis of the reaction: cis-2,3-dihydroxy-2,3-dihydro-p-cumate + NAD(+) = 2,3-dihydroxy-p-cumate + H(+) + NADH. | EC:1.3.1.58 |
| GO:0016156 |  | Catalysis of the reaction: NAD(+) + succinate = fumarate + H(+) + NADH. | EC:1.3.1.6 |
| GO:0018513 |  | Catalysis of the reaction: cis-1,2-dihydroxy-1,2-dihydrodibenzothiophene + NAD(+) = 1,2-dihydroxydibenzothiophene + H(+) + NADH. | EC:1.3.1.60 |
| GO:0018516 |  | Catalysis of the reaction: 4-chlorobenzoyl-CoA + chloride + NADP(+) = 2,4-dichlorobenzoyl-CoA + NADPH. | EC:1.3.1.63 |
| GO:0018517 |  | Catalysis of the reaction: cis-4,5-dihydroxycyclohexa-2,6-diene-1,2-dicarboxylate + NAD(+) = 4,5-dihydroxyphthalate + H(+) + NADH. | EC:1.3.1.64 |
| GO:0018518 |  | Catalysis of the reaction: 5,6-dihydroxy-3-methyl-5,6-dihydroquinolin-2(1H)-one + NAD(+) = 5,6-dihydroxy-3-methyl-2-oxo-1,2-dihydroquinoline + H(+) + NADH. | EC:1.3.1.65 |
| GO:0018519 |  | Catalysis of the reaction: cis-1,2-dihydro-3-ethylcatechol + NAD(+) = 3-ethylcatechol + H(+) + NADH. | EC:1.3.1.66 |
| GO:0018520 |  | Catalysis of the reaction: cis-1,2-dihydroxy-4-methylcyclohexa-3,5-diene-1-carboxylate + NADP+ = 4-methylcatechol + NADPH + H+ + CO2. | EC:1.3.1.67 |
| GO:0018521 |  | Catalysis of the reaction: 1,6-dihydroxy-2-methylcyclohexa-2,4-dienecarboxylate + NAD(+) = 3-methylcatechol + CO(2) + NADH. | EC:1.3.1.68 |
| GO:0050472 |  | Catalysis of the reaction: dihydrozeatin + NADP(+) = H(+) + NADPH + zeatin. | EC:1.3.1.69 |
| GO:0050092 |  | Catalysis of the reaction: (2R,3S)-tartrate + NAD(+) = dihydroxyfumarate + H(+) + NADH. | EC:1.3.1.7 |
| GO:0050615 |  | Catalysis of the reaction: 17-O-acetylnorajmaline + NADP(+) = 1,2-dihydrovomilenine + H(+) + NADPH. | EC:1.3.1.73 |
| GO:0032440 |  | Catalysis of the reaction: n-alkanal + NAD(P)+ = alk-2-enal + NAD(P)H + H+. | EC:1.3.1.74 |
| GO:0033728 |  | Catalysis of the reaction: chlorophyllide a + NADP+ = divinyl chlorophyllide a + NADPH + H+. | EC:1.3.1.75 |
| GO:0043115 |  | Catalysis of the reaction: NAD(+) + precorrin-2 = 2 H(+) + NADH + sirohydrochlorin. | EC:1.3.1.76 |
| GO:0033729 |  | Catalysis of the reaction: a flavan-3-ol + 2 NAD(P)+ = an anthocyanidin + 2 NAD(P)H + H+. | EC:1.3.1.77 |
| GO:0033730 |  | Catalysis of the reaction: L-arogenate + NADP+ = L-tyrosine + NADPH + CO2. | EC:1.3.1.78 |
| GO:0033731 |  | Catalysis of the reaction: L-arogenate + NAD(P)+ = L-tyrosine + NAD(P)H + CO2. | EC:1.3.1.79 |
| GO:0047616 |  | Catalysis of the reaction: acyl-CoA + NADP+ = 2,3-dehydroacyl-CoA + NADPH + H+. | EC:1.3.1.8 |
| GO:0052580 |  | Catalysis of the reaction: (-)-menthone + NADP+ = (+)-pulegone + NADPH + H+. | EC:1.3.1.81 |
| GO:0052581 |  | Catalysis of the reaction: (6R)-isoperitenone + H(+) + NADPH = (2R,5R)-isopulegone + NADP(+). | EC:1.3.1.82 |
| GO:0043957 |  | Catalysis of the reaction: acryloyl-CoA + NADPH + H+ = propionyl-CoA + NADP+. | EC:1.3.1.84 |
| GO:0016631 |  | Catalysis of the reaction: acyl-[acyl-carrier protein] + NAD(P)+ = trans-2,3-dehydroacyl-[acyl-carrier protein] + NAD(P)H + H+. | EC:1.3.1.9 |
| GO:0004085 |  | Catalysis of the reaction: butanoyl-CoA + electron-transfer flavoprotein = 2-butenoyl-CoA + reduced electron-transfer flavoprotein. | EC:1.3.8.1 |
| GO:0003960 |  | Catalysis of the reaction: NADPH + H+ + quinone = NADP+ + semiquinone. | EC:1.6.5.5 |
| GO:0052690 |  | Catalysis of the reaction: 2,3,6-trichlorohydroquinone + 2 glutathione = 2,6-dichlorohydroquinone + glutathione disulfide + HCl. | EC:1.6.99.1 |
| GO:0050446 |  | Catalysis of the reaction: N,N-dimethyl-1,4-phenylenediamine + aniline + NADP+ = 4-(dimethylamino)azobenzene + NADPH + H+. | EC:1.7.1.6 |
| GO:0047136 |  | Catalysis of the reaction: 4-(dimethylamino)azobenzene + H(2)O + NADP(+) = 4-(dimethylamino)phenylazoxybenzene + H(+) + NADPH. | EC:1.7.1.11 |
| GO:0047137 |  | Catalysis of the reaction: 2-acetamidofluorene + NAD(P)+ + H2O = N-hydroxy-2-acetamidofluorene + NAD(P)H + H+. | EC:1.7.1.12 |
| GO:0050629 |  | Catalysis of the reaction: trichloroethene + chloride + acceptor = tetrachloroethene + reduced acceptor. | EC:1.97.1.8 |
| GO:0030601 | Protease | OBSOLETE. Catalysis of the release of N-terminal Arg and Lys from oligopeptides when P1' is not Pro. Also acts on arylamides of Arg and Lys. | EC:3.4.11.6 |
| GO:0019131 |  | OBSOLETE. Catalysis of the release of an N-terminal tripeptide from a polypeptide; requires acid pH. | EC:3.4.14.9 |
| GO:0004188 |  | OBSOLETE. Catalysis of the cleavage of a Pro-Xaa bond by a serine-type peptidase mechanism to release a C-terminal amino acid. | EC:3.4.16.2 |
| GO:0004187 |  | OBSOLETE. Catalysis of the preferential release of a C-terminal arginine or lysine residue. Function is inhibited by diisopropyl fluorophosphate and sensitive to thiol-blocking reagents. | EC:3.4.16.6 |
| GO:0004263 |  | OBSOLETE. Catalysis of the preferential cleavage of Tyr-Xaa > Trp-Xaa > Phe-Xaa > Leu-Xaa. | EC:3.4.21.1 |
| GO:0004295 |  | OBSOLETE. Catalysis of the preferential cleavage of Arg-Xaa, Lys-Xaa. | EC:3.4.21.4 |
| GO:0004284 |  | OBSOLETE. Catalysis of the preferential cleavage of Arg-Xaa > Lys-Xaa. | EC:3.4.21.10 |
| GO:0003802 |  | OBSOLETE. Catalysis of the selective cleavage of one Arg-Ile bond in factor X to form factor Xa, and on factor IX to form factor IXa beta. | EC:3.4.21.21 |
| GO:0004287 |  | OBSOLETE. Catalysis of the hydrolysis of Pro-Xaa > Ala-Xaa in oligopeptides. | EC:3.4.21.26 |
| GO:0003805 |  | OBSOLETE. Catalysis of the selective cleavage of Arg-Ala and Arg-Val bonds in factor IX to form factor IXa. | EC:3.4.21.27 |
| GO:0004293 |  | OBSOLETE. Catalysis of the preferential cleavage of Arg-Xaa bonds in small molecule substrates. Highly selective action to release kallidin (lysyl-bradykinin) from kininogen involves hydrolysis of Met-Xaa or Leu-Xaa. The rat enzyme is unusual in liberating bradykinin directly from autologous kininogens by cleavage at two Arg-Xaa bonds. | EC:3.4.21.35 |
| GO:0030271 |  | OBSOLETE. Catalysis of the preferential cleavage: Phe-Xaa > Tyr-Xaa > Trp-Xaa > Leu-Xaa. | EC:3.4.21.39 |
| GO:0003815 |  | OBSOLETE. Catalysis of the selective cleavage of Lys(or Arg)-Ile bond in complement subcomponent C1s to form the active form of C1s (EC:3.4.21.42). | EC:3.4.21.41 |
| GO:0003816 |  | OBSOLETE. Catalysis of the cleavage of component C4 to C4a and C4b (Arg-Ala bond), and component C2 to C2a and C2b (Lys-Lys or Arg-Lys bond). | EC:3.4.21.42 |
| GO:0003817 |  | OBSOLETE. Catalysis of the cleavage of component factor B (Arg-Lys) when in complex with C3b or with cobra venom factor (CVF). | EC:3.4.21.46 |
| GO:0008846 |  | OBSOLETE. Catalysis of the hydrolysis of large proteins such as globin, casein and denatured serum albumin, in presence of ATP. | EC:3.4.21.53 |
| GO:0030019 |  | OBSOLETE. Catalysis of the preferential cleavage of Arg-Xaa, Lys-Xaa, but with more restricted specificity than trypsin. | EC:3.4.21.59 |
| GO:0004290 |  | OBSOLETE. Catalysis of the cleavage of the Arg-Xaa bond in Lys-Arg-Xaa and Arg-Arg-Xaa to process Yeast alpha-factor pheromone and killer toxin precursors. | EC:3.4.21.61 |
| GO:0004291 |  | OBSOLETE. Catalysis of the hydrolysis of proteins with broad specificity for peptide bonds, and a preference for a large uncharged residue in P1. Hydrolyzes peptide amides. | EC:3.4.21.62 |
| GO:0004276 |  | OBSOLETE. Catalysis of the release of mature proteins from their proproteins by cleavage of the terminal bond of Arg-Xaa-Yaa-Arg-Z motifs where Xaa can be any amino acid and Yaa is Arg or Lys. Releases albumin, complement component C3 and von Willebrand factor from their respective precursors. | EC:3.4.21.75 |
| GO:0004277 |  | OBSOLETE. Catalysis of the hydrolysis of proteins, including fibronectin, type IV collagen and nucleolin. Preferential cleavage: Arg-Xaa > Lys-Xaa > Phe-Xaa in small molecule substrates. | EC:3.4.21.78 |
| GO:0004278 |  | OBSOLETE. Catalysis of the preferential cleavage of Asp-Xaa > Asn-Xaa > Met-Xaa, Ser-Xaa. | EC:3.4.21.79 |
| GO:0008945 |  | OBSOLETE. Catalysis of the hydrolysis of Arg-Xaa and Lys-Xaa bonds in oligopeptides, even when P1' residue is proline. | EC:3.4.21.83 |
| GO:0009004 |  | OBSOLETE. Catalysis of the cleavage of N-terminal leader sequences from secreted and periplasmic proteins precursor. | EC:3.4.21.89 |
| GO:0008462 |  | OBSOLETE. Catalysis of the hydrolysis of proteins to small peptides in the presence of ATP and magnesium. Alpha-casein is the usual test substrate. In the absence of ATP, only oligopeptides shorter than five residues are cleaved, for example, succinyl-Leu-Tyr-NHMec which is cleaved at the Tyr-NHMec bond, and Leu-Tyr-Leu-Tyr-Trp which is cleaved at the second Leu-Typ bond (cleavage of the Tyr-Leu and Tyr-Trp bonds also occurs). | EC:3.4.21.92 |
| GO:0004285 |  | OBSOLETE. Catalysis of the release of protein hormones, neuropeptides and renin from their precursors, generally by cleavage of -Lys-Arg-Xaa at the Arg-Xaa bond. | EC:3.4.21.93 |
| GO:0019132 |  | OBSOLETE. Catalysis of the cleavage of peptides after specific recognition of a C-terminal tripeptide, Xaa-Yaa-Zaa, in which Xaa is preferably Ala or Leu, Yaa is preferably Ala or Tyr, and Zaa is preferably Ala. Cleavage is at a variable distance from the C-terminus; a typical cleavage is -Ala-Ala-Arg-Ala-Ala-Lys-Glu-Asn-Tyr-Ala-Leu-Ala-Ala. | EC:3.4.21.102 |
| GO:0051404 |  | OBSOLETE. Catalysis of the cleavage of Arg-Xaa bonds. | EC:3.4.22.8 |
| GO:0008129 |  | OBSOLETE. Catalysis of the hydrolysis of proteins with broad specificity for peptide bonds, with preference for a residue bearing a large hydrophobic side chain at the P2 position. Does not accept Val at P1'. | EC:3.4.22.14 |
| GO:0031079 |  | OBSOLETE. Catalysis of the selective cleavage of GlnGly bond in the poliovirus polyprotein. In other picornavirus reactions Glu may be substituted for Gln, and Ser or Thr for Gly. | EC:3.4.22.28 |
| GO:0001509 |  | OBSOLETE. Catalysis of the hydrolysis of proteins and small-molecule substrates at Asn-Xaa bonds. | EC:3.4.22.34 |
| GO:0008423 |  | OBSOLETE. Catalysis of the inactivation of bleomycin B2 (a cytotoxic glycometallopeptide) by hydrolysis of a peptide bond of beta-aminoalanine, but also shows general aminopeptidase activity. The specificity varies somewhat with source, but amino acid arylamides of Met, Leu and Ala are preferred. | EC:3.4.22.40 |
| GO:0046561 |  | OBSOLETE. Catalysis of the hydrolysis of proteins with broad specificity similar to that of pepsin A, preferring hydrophobic residues at P1 and P1', but also cleaving Gly20-Glu in the B chain of insulin. Clots milk, and activates trypsinogen. | EC:3.4.23.20 |
| GO:0004196 |  | OBSOLETE. Catalysis of the degradation of gelatin; little activity on hemoglobin. Specificity on B chain of insulin more restricted than pepsin A; does not cleave Phe1-Val2, Gln4-His5 or Gly23-Phe24. | EC:3.4.23.25 |
| GO:0017090 |  | A protein complex that is located in the cell membrane, and is involved in the metabolism of peptides, including neuropeptides. The complex has metalloendopeptidase activity that catalyzes the hydrolysis of protein and peptide substrates, preferentially on carboxyl side of hydrophobic residues. | EC:3.4.24.18 |
| GO:0004235 |  | OBSOLETE. Catalysis of the cleavage of Ala14-Leu15 and Tyr16-Leu17 in B chain of insulin. No action on collagen types I, II, IV and V. Cleaves gelatin chain alpha-2(I) > alpha-1(I). | EC:3.4.24.23 |
| GO:0004247 |  | OBSOLETE. Catalysis of the cleavage of Pro-Phe and Ala-Ala bonds. | EC:3.4.24.37 |
| GO:0008975 |  | OBSOLETE. Catalysis of the preferential cleavage of Tyr16-Leu17 and Phe25-Tyr26 bonds of oxidized insulin B chain. Also acts on other substrates of Molecular weight less than 7 kDa such as insulin and glucagon. | EC:3.4.24.55 |
| GO:0033264 |  | OBSOLETE. Catalysis of limited hydrolysis of proteins of the neuroexocytosis apparatus, synaptobrevins, SNAP25 or syntaxin. No detected action on small molecule substrates. | EC:3.4.24.69 |
| GO:0052577 | Terpene_Synthase | Catalysis of the reaction: 2-trans,6-trans-farnesyl diphosphate = (-)-germacrene D + diphosphate. | EC:4.2.3.22 |
| GO:0034005 |  | Catalysis of the reaction: 2-trans,6-trans-farnesyl diphosphate = (+)-(R)-germacrene A + diphosphate. | EC:4.2.3.23 |
| GO:0045482 |  | Catalysis of the reaction: 2-trans,6-trans-farnesyl diphosphate = diphosphate + trichodiene. | EC:4.2.3.6 |
| GO:0050467 |  | Catalysis of the reaction: 2-trans,6-trans-farnesyl diphosphate = diphosphate + pentalenene. | EC:4.2.3.7 |
| GO:0045483 |  | Catalysis of the reaction: trans,trans-farnesyl diphosphate = aristolochene + diphosphate. | EC:4.2.3.9 |
| GO:0050550 |  | Catalysis of the reaction: geranyl diphosphate = pinene + diphosphate. | EC:4.2.3.14 |
| GO:0052679 |  | Catalysis of the reaction: terpentedienyl diphosphate = diphosphate + terpentetriene. | EC:4.2.3.36 |
| GO:0052682 |  | Catalysis of the reaction: 2-trans,6-trans-farnesyl diphosphate + H2O = epi-cedrol + diphosphate. | EC:4.2.3.39 |
| GO:0047926 |  | Catalysis of the reaction: geranyl diphosphate = (2S)-bornyl diphosphate. | EC:5.5.1.8 |
| GO:0018697 | Nitrogenase | Catalysis of the reaction: carbonyl sulfide + 2 H+ + 2 e- = hydrogen sulfide + carbon monoxide. | EC:1.18.6.1 |
| GO:0050142 |  | Catalysis of the reaction: 6 reduced flavodoxin + 6 H+ + N2 + n ATP = 6 oxidized flavodoxin + 2 NH3 + n ADP + n phosphate. | EC:1.19.6.1 |
| GO:0080032 | Lipase | Catalysis of the reaction: a methyl jasmonate + H2O = a jasmonic acid + methanol. | EC:3.1.1.1 |
| GO:0004064 |  | Catalysis of the reaction: a phenyl acetate + H2O = a phenol + acetate. | EC:3.1.1.2 |
| GO:0050253 |  | Catalysis of the reaction: retinyl palmitate + H2O = retinol + palmitate + H+. | EC:3.1.1.3 |
| GO:0047499 |  | Catalysis of the reaction: phosphatidylcholine + H2O = 1-acylglycerophosphocholine + a carboxylate. This reaction does not require Ca2+. | EC:3.1.1.4 |
| GO:0004622 |  | Catalysis of the reaction: 2-lysophosphatidylcholine + H2O = glycerophosphocholine + a carboxylate. | EC:3.1.1.5 |
| GO:0004771 |  | Catalysis of the reaction: a steryl ester + H2O = a sterol + a fatty acid. | EC:3.1.1.13 |
| GO:0047372 |  | Catalysis of the reaction: H2O + acylglycerol = a fatty acid + glycerol. | EC:3.1.1.23 |
| GO:0047714 |  | Catalysis of the reaction: 1,2-diacyl-3-beta-D-galactosyl-sn-glycerol + 2 H2O = 3-beta-D-galactosyl-sn-glycerol + 2 carboxylates. | EC:3.1.1.26 |
| GO:0052740 |  | Catalysis of the reaction: 1-acyl-2-lysophosphatidylserine + H2O = sn-glycerol-phosphoserine + a carboxylate. | EC:3.1.1.32 |
| GO:0004465 |  | Catalysis of the reaction: triacylglycerol + H2O = diacylglycerol + a carboxylate, where the triacylglycerol is part of a lipoprotein. | EC:3.1.1.34 |
| GO:0003847 |  | Catalysis of the reaction: 2-acetyl-1-alkyl-sn-glycero-3-phosphocholine + H2O = 1-alkyl-sn-glycero-3-phosphocholine + acetate. | EC:3.1.1.47 |
| GO:0050185 |  | Catalysis of the reaction: 1-phosphatidyl-1D-myo-inositol + H(2)O = 1-acyl-sn-glycero-3-phospho-D-myo-inositol + a carboxylate + H(+). | EC:3.1.1.52 |
| GO:0033878 |  | Catalysis of the reactions: diacylglycerol + H2O = monoacylglycerol + a carboxylate; triacylglycerol + H2O = diacylglycerol + a carboxylate; and monoacylglycerol + H2O = glycerol + a carboxylate. | EC:3.1.1.79 |
| GO:0016290 |  | Catalysis of the reaction: palmitoyl-CoA + H2O = CoA + palmitate. | EC:3.1.2.2 |
| GO:0008474 |  | Catalysis of the reaction: palmitoyl-protein + H2O = palmitate + protein. | EC:3.1.2.22 |
| GO:0050429 |  | Catalysis of the reaction: a phosphatidylcholine + H2O = 1,2-diacylglycerol + choline phosphate. This reaction requires Ca2+. | EC:3.1.4.3 |
| GO:0070290 |  | Catalysis of the release of N-acylethanolamine from N-acyl-phosphatidylethanolamine (NAPE) to generate N-acylethanolamine (NAE). | EC:3.1.4.4 |
| GO:0004435 |  | Catalysis of the reaction: 1-phosphatidyl-1D-myo-inositol 4,5-bisphosphate + H(2)O = 1,2-diacylglycerol + 1D-myo-inositol 1,4,5-trisphosphate + H(+). | EC:3.1.4.11 |
| GO:0004767 |  | Catalysis of the reaction: H(2)O + sphingomyelin = ceramide + choline phosphate + H(+). | EC:3.1.4.12 |
| GO:0047391 |  | Catalysis of the reaction: H2O + 1-alkyl-sn-glycero-3-phosphoethanolamine = ethanolamine + 1-alkyl-sn-glycerol 3-phosphate. | EC:3.1.4.39 |
| GO:0050290 |  | Catalysis of the reaction: H(2)O + sphingomyelin = ceramide 1-phosphate + choline + H(+). | EC:3.1.4.41 |
| GO:0004621 |  | Catalysis of the reaction: glycoprotein phosphatidylinositol + H2O = phosphatidate + glycoprotein inositol. | EC:3.1.4.50 |
| GO:0004436 |  | Catalysis of the reaction: 1-phosphatidyl-1D-myo-inositol = D-myo-inositol 1,2-cyclic phosphate + diacylglycerol. | EC:4.6.1.13 |
| GO:0047396 |  | Catalysis of the reaction: 6-(alpha-D-glucosaminyl)-1-phosphatidyl-1D-myo-inositol = 1,2-diacyl-sn-glycerol + 6-(alpha-D-glucosaminyl)-1D-myo-inositol 1,2-cyclic phosphate. | EC:4.6.1.14 |
| GO:0004089 | CA | Catalysis of the reaction: H2CO3 = CO2 + H2O. | EC:4.2.1.1 |
| GO:0018818 | Acetylene hydratase | Catalysis of the reaction: acetaldehyde = acetylene + H(2)O. | EC:4.2.1.112 |
| GO:0043758 | AcetylCoA_Synthetase | Catalysis of the reaction: ATP + acetate + CoA = ADP + phosphate + acetyl-CoA. | EC:6.2.1.13 |
| GO:0030729 |  | Catalysis of the reaction: acetoacetate + ATP + CoA = acetoacetyl-CoA + AMP + diphosphate + H(+). | EC:6.2.1.16 |
| GO:0015250 | Aquaporin | Transport systems of this type catalyze facilitated diffusion of water (by an energy-independent process) by passage through a transmembrane aqueous pore or channel without evidence for a carrier-mediated mechanism. | BRENDA name search |
| GO:0006833 |  | The directed movement of water (H2O) into, out of or within a cell, or between cells, by means of some agent such as a transporter or pore. | BRENDA name search |
