# Supplementary material for: Mining a database of single amplified genomes from Red Sea brine pool extremophiles—improving reliability of gene function prediction using a profile and pattern matching algorithm (PPMA)
Source: Front Microbiol. 2014 Apr 7;5:134. doi: 10.3389/fmicb.2014.00134 (PMC3985023; doi:10.3389/fmicb.2014.00134)
Supplement: Supplementary file 5 [file DataSheet5.DOCX]

**Table S4. The 49 consensus pattern used fort he pattern analysis.**

| **Type** | **Consensus pattern** | **Cons. pattern - Info** | **PROSITE ID** |
| --- | --- | --- | --- |
| ADH | [STALIV]-[LIVF]-x-[DE]-x(6,7)-P-x(4)-[ALIV]-x-[GST]-x(2)-D-[TAIVM]-[LIVMF]-x(4)-E | ADH_IRON_1 | PS00913 |
|  | [GSW]-x-[LIVTSACD]-[GH]-x(2)-[GSAE]-[GSHYQ]-x-[LIVTP]-[GAST]-[GAS]-x(3)-[LIVMT]-x-[HNS]-[GA]-x-[GTAC] | ADH_IRON_2 | PS00060 |
|  | [LIVSPADNK]-x(9)-{P}-x(2)-Y-[PSTAGNCV]-[STAGNQCIVM]-[STAGC]-K-{PC}-[SAGFYR]-[LIVMSTAGD]-x-{K}-[LIVMFYW]-{D}-x-{YR}-[LIVMFYWGAPTHQ]-[GSACQRHM] | ADH_SHORT | PS00061 |
|  | G-H-E-x-{EL}-G-{AP}-x(4)-[GA]-x(2)-[IVSAC] | ADH_ZINC | PS00059 |
|  | [GSDN]-[DEQHKM]-x(2)-L-x(3)-[SAG](2)-G(2)-x-G-x(4)-Q-x(2)-[KRS] | QOR_ZETA_CRYSTAL | PS01162 |
|  | G-[FY]-R-[HSAL]-[LIVMF]-D-[STAGCL]-[AS]-x(5)-[EQ]-x(2)-[LIVMCA]-[GS] | ALDOKETO_REDUCTASE_1 | PS00798 |
|  | [LIVMFY]-x(8)-{L}-[KREQ]-{K}-[LIVM]-G-[LIVM]-[SC]-N-[FY] | ALDOKETO_REDUCTASE_2 | PS00062 |
|  | [LIVM]-[PAIV]-[KR]-[ST]-{EPQG}-{RFI}-x(2)-R-{SVAF}-x-[GSTAEQK]-[NSL]-x-{LVRI}-[LIVMFA] | ALDOKETO_REDUCTASE_3 | PS00063 |
| Formate DH | [LIVMA]-[AG]-[IVT]-[LIVMFY]-[AG]-x-G-[NHKRQGSAC]-[LIV]-G-x(13,14)-[LIVMFT]-{A}-x-[FYWCTH]-[DNSTK] | D_2_HYDROXYACID_DH_1 | PS00065 |
|  | [LIVMFYWA]-[LIVFYWC]-x(2)-[SAC]-[DNQHR]-[IVFA]-[LIVF]-x-[LIVF]-[HNI]-x-P-x(4)-[STN]-x(2)-[LIVMF]-x-[GSDN] | D_2_HYDROXYACID_DH_2 | PS00670 |
|  | [LMFATCYV]-[KPQNHAR]-x-[GSTDNK]-x-[LIVMFYWRC]-[LIVMFYW](2)-N-x-[STAGC]-R-[GP]-x-[LIVH]-[LIVMCT]-[DNVE] | D_2_HYDROXYACID_DH_3 | PS00671 |
|  | [STAN]-x-[CH]-x(2,3)-C-[STAG]-[GSTVMF]-x-C-x-[LIVMFYW]-x-[LIVMA]-x(3,4)-[DENQKHT] | MOLYBDOPTERIN_PROK_1 | PS00551 |
|  | [STA]-x-[STAC](2)-x(2)-[STA]-D-[LIVMY](2)-L-P-x-[STAC](2)-x(2)-E | MOLYBDOPTERIN_PROK_2 | PS00490 |
|  | A-x(3)-[GDTN]-[IF]-x-[DNQTKEH]-x-[DEAQ]-x-[LIVM]-x-[LIVMC]-x-[NS]-x(2)-[GS]-x(4,5)-[AV]-x-[LIVMEF]-[STY] | MOLYBDOPTERIN_PROK_3 | PS00932 |
| Ene-Re-duc-tase | [ERND]-[IVL]-x-[ED]-x-H-x(3)-K-x-[DE]-x(2)-S-[GA]-[TAS]-[ALCGS] | DAPB | PS01298 |
| Protease | [HA]-[GSYR]-[LIVMT]-[SG]-H-x-[LIV]-G-[LIVMNKS]-x-[IVEL]-[HNC]-[DEV] | PROLINE_PEPTIDASE | PS00491 |
|  | [LIVF]-x(2)-[LIVSTA]-x-[IVPST]-x-[GSDNQL]-[SAGV]-[SG]-H-x-[IVAQ]-P-x(3)-[PSA] | CARBOXYPEPT_SER_HIS | PS00560 |
|  | [LIVM]-x-[GSTA]-E-S-Y-[AG]-[GS] | CARBOXYPEPT_SER_SER | PS00131 |
|  | Q-x(3)-N-[SA]-C-G-x(3)-[LIVM](2)-H-[SA]-[LIVM]-[SA] | UCH_1 | PS00140 |
|  | D-x(3)-A-x(3)-[LIVMFYW]-x(14)-G-x-S-x-G-G-[LIVMFYW](2) | PRO_ENDOPEP_SER | PS00708 |
|  | C-x(3)-[LIVMFY]-x(5)-[LIVMFY]-x(3)-[DENQ]-[LIVMFY]-x(10)-C-x(3)-C-T-x(4)-C-x-[LIVMFY]-F-x-[FY]-x(13,14)-C-x-[LIVMFY]-[RK]-x-[ST]-x(14,15)-S-G-x-[ST]-[LIVMFY]-x(2)-C | APPLE | PS00495 |
|  | D-G-[PD]-S-A-[GS]-[LIVMCA]-[TA]-[LIVM] | LON_SER | PS01046 |
|  | [STAIV]-{ERDL}-[LIVMF]-[LIVM]-D-[DSTA]-G-[LIVMFC]-x(2,3)-[DNH] | SUBTILASE_ASP | PS00136 |
|  | H-G-[STM]-x-[VIC]-[STAGC]-[GS]-x-[LIVMA]-[STAGCLV]-[SAGM] | SUBTILASE_HIS | PS00137 |
|  | G-T-S-x-[SA]-x-P-x-{L}-[STAVC]-[AG] | SUBTILASE_SER | PS00138 |
|  | [GS]-{PR}-S-M-{RS}-[PS]-[AT]-[LF] | SPASE_I_1 | PS00501 |
|  | K-R-[LIVMSTA](2)-[GA]-x-[PG]-G-[DEQ]-x-[LIVM]-x-[LIVMFY] | SPASE_I_2 | PS00760 |
|  | [LIVMFYW](2)-{NLPA}-{T}-G-D-[NH]-{PIEW}-x(2)-[SND]-x(2)-[SG] | SPASE_I_3 | PS00761 |
|  | R-x(3)-[EAP]-x(3)-[LIVMFYT]-[LM]-[LIVM]-H-Q-P | CLP_PROTEASE_HIS | PS00382 |
|  | T-x(2)-[LIVMF]-G-x-A-[SAC]-S-[MSA]-[PAG]-[STA] | CLP_PROTEASE_SER | PS00381 |
|  | Q-{V}-x-{DE}-[GE]-{F}-C-[YW]-{DN}-x-[STAGC]-[STAGCV] | THIOL_PROTEASE_CYS | PS00139 |
|  | [LIVMFGAC]-[LIVMTADN]-[LIVFSA]-D-[ST]-G-[STAV]-[STAPDENQ]-{GQ}-[LIVMFSTNC]-{EGK}-[LIVMFGTA] | ASP_PROTEASE | PS00141 |
|  | [GSTALIVN]-{PCHR}-{KND}-H-E-[LIVMFYW]-{DEHRKP}-H-{EKPC}-[LIVMFYWGSPQ] | ZINC_PROTEASE | PS00142 |
|  | P-R-C-[GN]-x-P-[DR]-[LIVSAPKQ] | CYSTEINE_SWITCH | PS00546 |
|  | D-x-[LI]-x(4)-G-x-D-x-[LI]-x-G-G-x(3)-D | HEMOLYSIN_CALCIUM | PS00330 |
| Nitrogenase | E-x-G-G-P-x(2)-[GA]-x-G-C-[AG]-G | NIFH_FRXC_1 | PS00746 |
|  | D-x-L-G-D-V-V-C-G-G-F-[AGSP]-x-P | NIFH_FRXC_2 | PS00692 |
|  | [LIVMFYH]-[LIVMFST]-H-[AG]-[AGSP]-[LIVMNQA]-[AG]-C | NITROGENASE_1_1 | PS00699 |
|  | [STANQ]-[ET]-C-x(5)-G-D-[DN]-[LIVMT]-x-[STAGR]-[LIVMFYST] | NITROGENASE_1_2 | PS00090 |
| Lipase | F-[GR]-G-x(4)-[LIVM]-x-[LIV]-x-G-x-S-[STAG]-G | CARBOXYLESTERASE_B_1 | PS00122 |
|  | [EDA]-[DG]-C-L-[YTF]-[LIVT]-[DNS]-[LIV]-[LIVFYW]-x-[PQR] | CARBOXYLESTERASE_B_2 | PS00941 |
|  | [LIV]-{KG}-[LIVFY]-[LIVMST]-G-[HYWV]-S-{YAG}-G-[GSTAC] | LIPASE_SER | PS00120 |
|  | [LIVMA]-C-{LIVMFYWPCST}-C-D-{GS}-{G}-{N}-x-{QS}-C | PA2_ASP | PS00119 |
|  | C-C-{P}-x-H-{LGY}-x-C | PA2_HIS | PS00118 |
|  | H-Y-x-[GT]-D-[LIVMAF]-[DNSH]-x-P-x-H-[PA]-x-N | PROKAR_ZN_DEPEND_PLPC_1 | PS00384 |
| Carbonic Anhydrase | S-E-[HN]-x-[LIVM]-x(4)-[FYH]-x(2)-E-[LIVMGA]-H-[LIVMFA](2) | ALPHA_CA_1 | PS00162 |
|  | C-[SA]-D-S-R-[LIVM]-x-[AP] | PROK_CO2_ANHYDRASE_1 | PS00704 |
|  | [EQ]-[YF]-A-[LIVM]-x(2)-[LIVM]-x(4)-[LIVMF](3)-x-G-H-x(2)-C-G | PROK_CO2_ANHYDRASE_2 | PS00705 |
| Aquaporin | [HNQA]-{D}-N-P-[STA]-[LIVMF]-[ST]-[LIVMF]-[GSTAFY] | MIP family signature | PS00221 |
